# Working Memory Training in Post-Secondary Students with ADHD: A Randomized Controlled Study

**DOI:** 10.1371/journal.pone.0137173

**Published:** 2015-09-23

**Authors:** Karizma Mawjee, Steven Woltering, Rosemary Tannock

**Affiliations:** 1 University of Toronto, Toronto, Ontario, Canada; 2 Texas A&M University, College Station, Texas, United States of America; 3 Hospital for Sick Children, Toronto, Ontario, Canada; Philipps University Marburg, GERMANY

## Abstract

**Objectives:**

To determine whether standard-length computerized training enhances working memory (WM), transfers to other cognitive domains and shows sustained effects, when controlling for motivation, engagement, and expectancy.

**Methods:**

97 post-secondary students (59.8% female) aged 18–35 years with Attention-Deficit/Hyperactivity Disorder, were randomized into standard-length adaptive Cogmed WM training (CWMT; 45-min/session), a shortened-length adaptive version of CWMT (15 min/session) that controlled for motivation, engagement and expectancy of change, or into a no training group (waitlist-control group). All three groups received weekly telephone calls from trained coaches, who supervised the CWMT and were independent from the research team. All were evaluated before and 3 weeks post-training; those in the two CWMT groups were also assessed 3 months post-training. Untrained outcome measures of WM included the WAIS-IV Digit Span (auditory-verbal WM), CANTAB Spatial Span (visual-spatial WM) and WRAML Finger Windows (visual-spatial WM). Transfer-of-training effects included measures of short-term memory, cognitive speed, math and reading fluency, complex reasoning, and ADHD symptoms.

**Results:**

Performance on 5/7 criterion measures indicated that shortened-length CWMT conferred as much benefit on WM performance as did standard-length training, with both CWMT groups improving more than the waitlist-control group. Only 2 of these findings remained robust after correcting for multiple comparisons. Follow-up analyses revealed that post-training improvements on WM performance were maintained for at least three months. There was no evidence of any transfer effects but the standard-length group showed improvement in task-specific strategy use.

**Conclusions:**

This study failed to find robust evidence of benefits of standard-length CWMT for improving WM in college students with ADHD and the overall pattern of findings raise questions about the specificity of training effects.

**Trial Registration:**

ClinicalTrials.gov NCT01657721

## Introduction

Computer-based cognitive training has emerged as a novel, non-invasive treatment option for training working memory (WM). WM refers to the ability to hold and manipulate relevant information in mind for a few seconds in the face of distracting or intrusive thoughts [1]. There are marked individual differences in WM capacity and these differences have been found to correlate positively with individual differences in high-level cognitive abilities, such as reading, mathematics, reasoning, sustaining attention, and fluid intelligence [2]. WM is reported to be impaired in many clinical disorders, including Attention-Deficit/Hyperactivity Disorder (ADHD), which is the population under investigation in the present study [3,4].

Given the documented associations between WM, high-level cognitive abilities, a variety of cognitive training paradigms have been developed with the goal of improving working memory. The rationale is that if it is possible to increase a person’s WM, then improvement should also occur in performance on other cognitive abilities that are strongly related to WM [5,6]. However, it is important to acknowledge that most of the evidence of associations between WM and other cognitive and academic outcomes, as well as between WM and ADHD, is correlational in nature. Thus, WM may be linked indirectly to these variables and not necessarily part of the causal pathway, in which case WM training would not be expected to transfer to other cognitive abilities or behavioral symptoms of ADHD [7,8]. Nonetheless, whether WM capacity itself can be improved is an issue of both theoretical and clinical importance.

CogMed Working Memory Training (CMWT) is one such program that claims to improve working memory [9, 10]. Designed initially to improve the documented visuospatial span deficits in WM in children with ADHD [11, 12], the standard CWMT program uses an adaptive algorithm that continually adjusts task difficulty on a trial-by-trial basis to match the individual’s current WM capacity to ensure that the trainee is always maximally challenged. CWMT involves intensive daily practice (30–45 min per day) for 5 days per week for 5 weeks, during which trainees repeatedly perform WM tasks, with feedback and rewards based on the accuracy for every trial. Training activities, which are based on Baddeley’s multi-componential model of WM [1], involve primarily simple visual-spatial span tasks, with the minority involving more complex activities requiring the executive component of WM. Since no WM theory predicts changes in WM capacity following training, CWMT’s premise that intensive training will increase working memory capacity appears to be based loosely on hypotheses about brain plasticity [9, 13].

Initial studies of CWMT were promising: they not only found improvements in WM but also in other related cognitive functions, as well as a reduction in the behavioral symptoms of ADHD, with effects lasting as long as 6-months post intervention [11, 14]. By contrast, recent meta-analyses have concluded that cognitive training produces only short-term effects on tasks similar to those used during training [15–17]. However, these meta-analyses aggregated effect sizes from studies using different training programs and diverse control groups. To date, there are 18 published studies investigating the effects of CWMT on individuals with ADHD, of which 10 are randomized controlled trials (RCTs). Most of these RCTs compared the standard adaptive version of CWMT with either its active but non-adaptive version or with a passive wait-list control group [cogmed web site, accessed June 22 2015]. Neither of these comparison groups provides adequate control for non-specific effects of training, such as the participant’s level of arousal, engagement, motivation, and expectancy for change [7].

Accordingly the primary aim of the present study was to determine whether the standard-length CWMT program enhances working memory when controlling for non-specific effects of engagement, motivation, and expectancy of improvement. To do so, we used three arms; 1) one treatment arm, in which participants received standard-length CWMT (25 daily training sessions of 45-minutes over a 5-week period and weekly calls from a certified CWMT coach), 2) an active control arm, in which participants received a ‘low dose’ of adaptive training (i.e., a shortened adaptive version of CWMT that also involved weekly coach-calls over a 5 week period, but the daily training sessions were shortened to 15-minutes), and 3) a no-training arm (wait-list control), in which participants received weekly coach-calls to control for possible effects of coaching (e.g., personal attention and encouragement), but did not undergo any working memory training. A second aim was to determine whether CWMT would transfer to untrained working memory tasks and domains other than working memory. The third aim was to determine whether any training effects persisted or emerged several months after training was completed. There are several reasons why the use of an adaptive ‘low dose CWMT’ would control for non-specific effects of training. First, the adaptive training simulates levels of arousal and engagement akin to that associated with standard training. Second, participants are exposed to identical training exercises, lending credence to their subjective belief that this training will benefit working memory. Third, the use of identical exercises for the low dose control group, as opposed to substitute ones, also precludes the acquisition of different skills that may inadvertently influence outcome measures (e.g., a racing game may train visuospatial skills) and they also enforce the subjective belief that training involved cognitive domains that required improvement. Although a low-dose control group does not control for total training time, and provides only a partial test for ‘dose-effects’ of WM training (a comprehensive test would require at least three dose levels), its design poses substantial advantages over other types of ‘active’ control groups.

**Fig 1:**
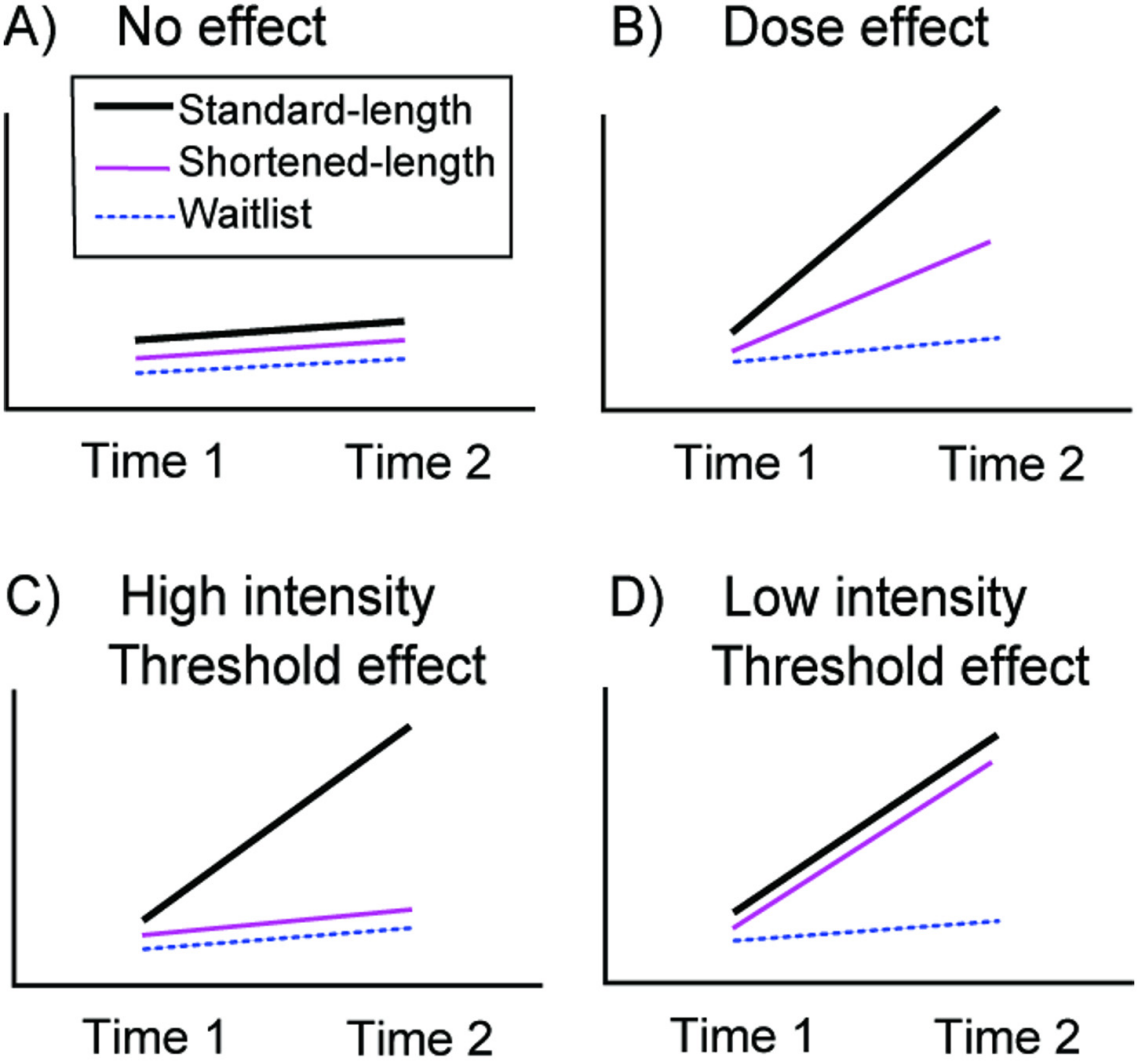
Hypothetical Effects.

Four hypothetical scenarios for results for our RCT are shown in Fig 1. Findings conforming to the pattern shown in Panel A would indicate no effects of working memory training. The pattern of findings shown in Panel B indicates a classic ‘dose-effect’, in which shortened-length training confers some benefits, but not as great as with standard-length training. In contrast, Panel C shows a high-intensity threshold effect, in which only the standard-length training confers beneficial effects, with shortened-length training being no different than waitlist control. The fourth panel, Panel D indicates a low-intensity threshold effect in which shortened-length training confers as much benefit as standard-length training.

Based on CWMT goal and theory, for criterion and near-transfer measures we predicted either a dose effect (Panel B in Fig 1: standard-length training > shortened-length training > waitlist control) or a high-intensity threshold effect (Panel C: standard-length training > shortened-length training = waitlist control) at post-treatment. For far transfer measures we predicted a high intensity threshold effect (Panel C: standard-length training > shortened-length training = waitlist control) at post-treatment. We also predicted a high-intensity threshold effect for sustained benefits at follow-up.

We conducted the study with post-secondary students diagnosed with ADHD, a subpopulation of emerging adults with ADHD, who, despite their high functioning, manifest more difficulties with concentration, time management skills, academics and WM than do their non-disordered peers [18–19]. Moreover, the use of young adults who were responsible for their own training and self-reinforcement eliminates potential effects that might arise from other’ s supervision of CWMT (e.g., supervision and reinforcement of children by parents, teachers, research assistants).

## Methods

### 2.1 Participants

A total of 97 post-secondary students diagnosed with ADHD (59.8% female), aged 18–35 (*M* = 23.9, *SD* = 3.41), participated in this study (see Table 1). Students were recruited through list-serv emails sent from Student Disability Services exclusively to students registered with ADHD. In Canada, registration with Student Disability Services requires students to provide comprehensive documentation to confirm their diagnosis or undergo a new diagnostic assessment. In our sample, 22 of the students (22.7%) also self-reported a comorbid learning disability. Of the 50 (51.5%) participants who were being treated with medication for their ADHD symptoms, 6 received Ritalin, 17 received Concerta, 11 received Vyvanse, 9 received Adderal, 3 received Dexadrine, 3 received Stratterra and 1 received another medication. Medication status was not controlled in this study, but participants were advised to maintain their current pharmacological treatment throughout the study. Medication status and dose were recorded at each visit: At T2, in the standard-length group two participants changed their medication, in the shortened-length group two participants changed their medication and one decreased their dose and in the waitlist control group 1 participant changed their medication.

**Table 1:** Participant Characteristics.

	Standard-length group (n = 32)		Shortened-length group (n = 33)		Waitlist Control Group (n = 32)		Total (n = 97)		*Group Differences (p-values)*
	*M*	*SD*	*M*	*SD*	*M*	*SD*	*M*	*SD*	
Gender									
Males (n)	13		12		14		39		.83
Females (n)	19		21		18		58		
School									
University	23		28		23		74		.36
College	9		5		9		23		
Self-reported learning difficulties	8		8		6		22		.78
Medication	19		18		19		56		.30
Age (year)	23.78	2.90	24.27	3.56	23.53	3.78	23.87	3.41	.68
ASRS-A Interview^a^	17.94	2.26	17.45	2.20	16.93	2.73	17.45	2.41	.70
ASRS-A Symptom Count^a^	4.97	.82	5.03	.77	4.87	.73	4.96	.77	.98
ASRS T1 Symptom Count^a^	5.03	.79	5.15	.83	4.97	.97	5.05	.86	.69
ASRS T1 Total^a^	49.00	9.65	48.67	8.15	49.19	10.78	48.95	9.47	.98
ASRS Other^a^	43.89	11.75	46.76	14.05	41.50	7.78	45.08	12.54	.74
ASRS Inattentive Symptoms^a^	27.03	4.56	26.00	3.84	26.81	5.05	26.6	4.48	.62
ASRS Hyperactive Symptoms^a^	22.00	6.31	22.94	5.29	22.38	7.21	22.44	5.25	.83
Symptom Assessment 45^e^	60.50	6.51	59.91	8.42	60.06	7.45	60.15	7.44	.054
GRIT^a^	30.03	6.16	31.91	6.20	31.44	6.33	31.13	6.22	.46
WASI IQ^b^	115.25	9.86	109.52	10.52	109.47	14.51	111.39	11.98	.08
TOWRE^b^	105.69	13.07	107.03	10.67	105.56	10.56	106.10	11.38	.85
Woodcock Johnson Math Fluency^b^	93.32	10.21	92.61	11.59	91.97	13.31	92.63	11.67	.90
WAIS Digit Span^b^	8.81	3.02	9.38	3.17	8.56	2.51	8.92	2.90	.52
CANTAB Spatial Span Backwards^c^	-.39	.94	.079	1.27	-.48	1.13	-.26	1.14	.12
CANTAB Spatial Span Forwards^c^	.087	1.16	.34	1.12	.054	1.27	.10	1.16	.23
CANTAB Pattern Recognition Memory^c^	-.069	.93	-.032	.90	-.11	.93	-.069	.91	.95
CANTAB WM Between Errors Score^c^	-.32	1.27	.17	1.10	-.30	1.19	-.15	1.20	.18
CANTAB WM Strategy Score^c^	-.11	1.08	.34	1.12	.054	1.27	.097	1.16	.28
WRAML Finger Windows Forwards^b^	9.47	4.20	11.42	3.07	9.16	3.57	10.03	3.74	.028
Cognitive Failures Questionnaire^a^	57.69	11.62	58.00	13.02	58.72	16.45	58.13	13.64	.96
BDEFS^d^	94.77	7.19	92.13	12.00	94.65	7.77	93.83	9.26	.44

^a^. Raw scores

^b^. Standardized scores

^c^. Z-score

^d^. Percentile Rank

^e^. T-score. NOTE: ASRS = ADHD Self-Report Scale; WASI = Wechsler Abbreviated Scale of Intelligence; TOWRE = Test of Word Reading Efficiency; WAIS = Wechsler Adult Intelligence Scale; CANTAB = Cambridge Neuropsychological Testing Automated Battery; WRAML = Wide Range Assessment of Memory and Learning; BDEFS = Barkley Deficits in Executive Functioning Scale.

Semi-structured telephone interviews were conducted to assess students’ eligibility to participate in the study as well as validate current ADHD symptomology. Inclusion criteria were as follows: 1) previous diagnosis of ADHD, 2) between 18 to 35 years of age 3) current enrollment in a post-secondary educational institution, 4) registered with Student Accessibility/Disability Services with a confirmed diagnosis of ADHD, 5) current symptoms consistent with diagnostic criteria for ADHD, as indicated by a semi-structured telephone interview based on the first six-items of the Adult ADHD Self-Report Scale (ASRS-A Interview), meeting the clinical cut-off score on the 18-item paper-version of the ASRS (ASRS T1), and on a collateral report using the adapted 18-item version of Adult ASRS completed by a significant other (ASRS Other) [20–21]. In addition to the total scores for the different versions of the ASRS, the scale is also used to calculate a ‘symptom count’; a score of ≥2 (sometimes, often, very often) on Items 1 to 3, and ≥3 (often, very often) on Items 4 to 6, with at least 4/ 6 items meeting these criteria, indicates a current symptom profile consistent with a diagnosis of ADHD. We have previously demonstrated the utility and reliability of these methods in confirming current symptoms and impairment in college students with ADHD [20, 21]. Exclusion criteria included: 1) major neurological dysfunction or psychosis, 2) current use of sedating or mood-altering medication other than medication provided for ADHD, 3) uncorrected sensory impairment, 4) motor or perceptual handicap that would prevent use of a computer program, or 5) a history of concussion or traumatic brain injury prior to ADHD diagnosis, 6) limited proficiency in English language. Exclusion criteria were ascertained from self-report during the intake interview.

**Fig 2:**
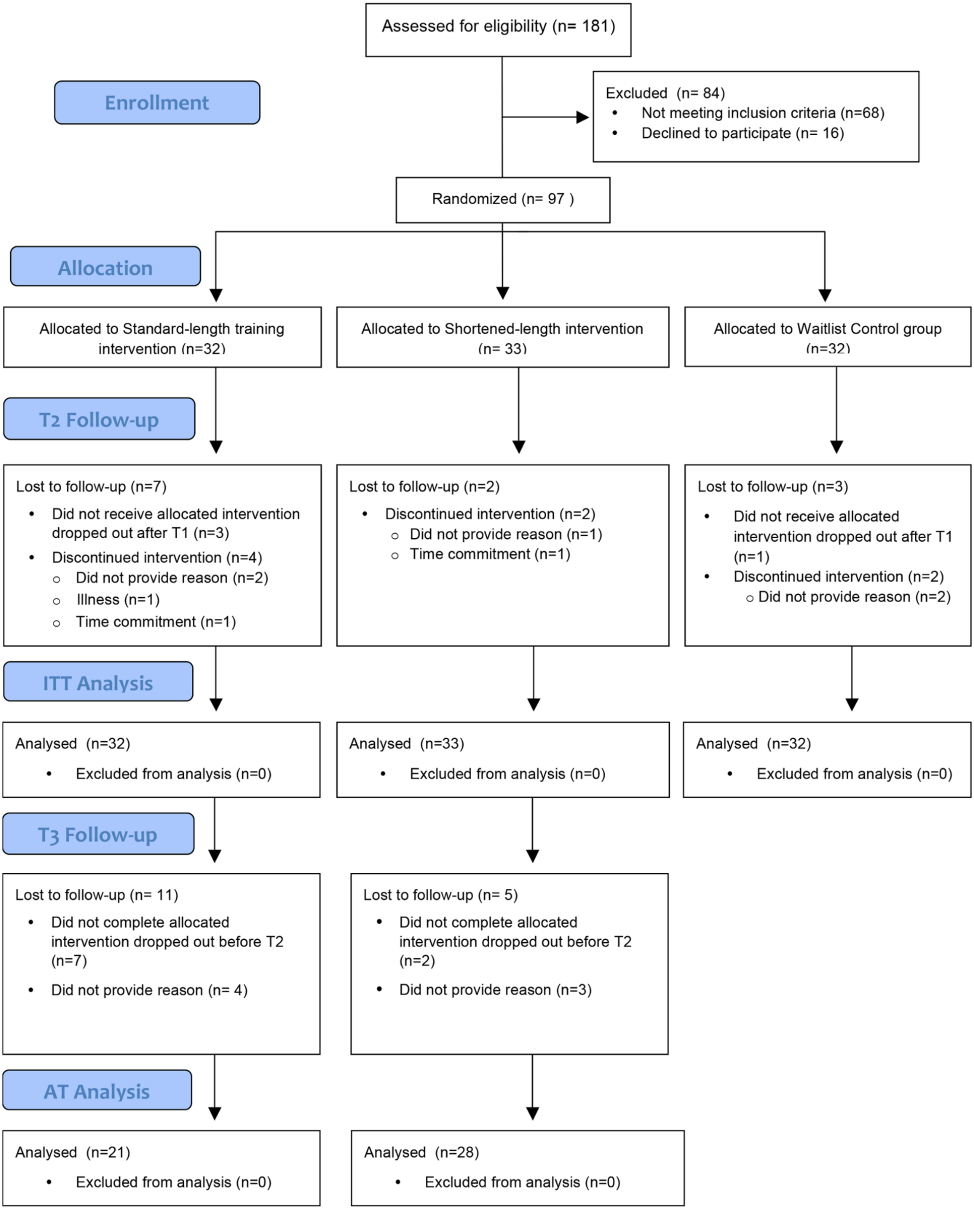
CONSORT Diagram.

Eligible participants were randomized into one of three arms: standard-length training with 45 minute-sessions (standard-length training; n = 32), shortened-length training with 15 minute-sessions (shortened-length training; n = 33) or a delayed-training waitlist-control group (waitlist control; n = 32). See Fig 2 for CONSORT diagram of participant flow through the study (S1 CONSORT Checklist). Baseline values for age, IQ, severity of ADHD symptoms, psychiatric symptomatology, comorbid learning difficulties, math or reading fluency, or perseverance of effort (as measured by the GRIT, described later) did not differ amongst the treatment groups. Demographic and descriptive characteristics of each treatment arm are shown in Table 1.

### 2.2 Intervention Program

The CWMT program, developed by CogMed Cognitive Medical Systems AB (Stockholm, Sweden), was selected as the intervention program in this study, since it has the most empirical evidence to support its efficacy in improving working memory [3]. The intervention program and coach-calls (described below) were provided by a licensed community psychology-services agency, independent of the research team. Based on an initial survey of participants’ preference, as well as its demonstrated utility in our pilot study [22], we used the school-aged RM version of the CWMT online-based computer program, implemented through the company’s website (www.cogmed.com) and completed in participants’ homes or place of residence. The RM version is almost identical to the adult QM version, with the same exercises and daily training times, but the user interface is more colorful and visually pleasing than the QM version, and also includes a RoboRacing game as an incentive and reinforcement for staying on task [10].

The standard RM version of CWMT consists of 12 auditory-verbal and visual-spatial working memory tasks that involve the storage and manipulation of particular sequences of stimuli. For each task, an adaptive algorithm automatically adjusts the difficulty level based on trial-by-trial performance to ensure individuals are always working at the upper limit of their working memory capacity. Positive reinforcement is provided at the end of each trial through computerized verbal feedback. The program requires 25 training sessions, typically described as taking about 45 minutes per session, to be completed 5 days per week, for 5–6 weeks. Weekly telephone calls from a certified CWMT coach are conducted to provide feedback on training performance, address any training challenges, make recommendations for the next week of training, and encourage compliance with the training schedule.

In this study, participants in the standard-length training group engaged in 45 minutes of training, completing 2 ‘core’ training activities (Visual Data Link and Input Module, S1 Table) per session that were used throughout training plus another 6 of the remaining 10 possible tasks per session, which were chosen based on random computer selection of tasks, for a total of 90 working memory trials. Those in the shortened-length training group engaged in 15 minutes of training, completing 45 trials of 4 working memory tasks per session, which consisted of the same two ‘core’ activities used throughout training (as in the standard-length program) plus two additional tasks that changed during each training session based on random computer selection. This shortened-length version was developed by Cogmed at the request of consumers who had completed the standard 25 days of WM training to provide ‘extension training’, but the company acknowledges that it has no research basis as yet (http://www.cogmed.com/questions-answers-training-of-working-memory-in-children-with-attention-deficits).

Participants were requested to engage in five training sessions (one session per day of the specified length) per week, for 5 weeks. They were advised to try to do the training at the same time each day, Monday to Friday and take a break from training over the weekend. Both CMWT programs employed an adaptive algorithm to adjust task level difficulty, and both CWMT groups received five 30-minute coach-calls (one call per week). Coaches encouraged participants in both groups to choose and implement incentives for self-reinforcement after completing each training session. Thus, the training groups differed only in the duration of the daily training sessions and number of different training activities per session.

Waitlist control participants did not undergo any training during the 5-week period, but did receive weekly calls from a certified CWMT coach to control for possible effects of coaching, attention and motivation, with each call lasting approximately 30 minutes. Coach-calls to waitlist control participants, which were a unique component of this study, covered five topics for which the students were provided website resources: Understanding ADHD (www.caddac.ca), Guidelines for post-secondary students with ADHD (www.caddac.ca, www.totallyaddconnect.com), Tips for managing ADHD at college (www.totallyadhdconnect.com/forums/tools), Explore working memory (www.teachadhd.ca), and Explore coaching for ADHD (www.teachadhd.ca, www.caddac.ca/resources/coaches). After their T2 assessment, waitlist control participants were able to choose between the standard-length and shortened-length working memory training.

### 2.3 Procedure

This study was approved by the Institutional Research Ethics Boards at the participating post-secondary institutions, as well as by the community agency providing the working memory training program. Informed written consent was obtained from all participants prior to entering the study (S1 Protocol). Prior to commencing the study, we had conducted a smaller-scale pilot study of CWMT to determine an appropriate sample size and evaluate the potential utility of shortened-length training, as well as other study components, such as recruitment procedures, intake criteria, coach calls and other aspects of the general protocol to aid in design refinements for this larger RCT [22]. Separate samples were recruited for the pilot and current study.

Participants in the standard-length training and shortened-length training groups were assessed individually at three different time points; prior to training (T1), and 3 weeks after completing training (T2) and three months after completing training (T3). Participants in the waitlist control group were assessed individually prior to training (T1) and 3 weeks after completing training (T2): they did not undergo assessment at T3 as we were obligated to offer them access to the training program while they were enrolled in post-secondary education. Due to the different nature of the three treatment arms it was not possible to keep participants, assessors, or CWMT Coaches blind as to which group participants had been randomized. However, results of the randomization were not revealed to participants until after the initial assessment.

During each assessment, participants completed a behavioral assessment, which included a battery of neuropsychological tests and behavioral rating scales, and a neural assessment, in which they underwent electroencephalography (EEG) while completing a battery of executive function tasks (e.g., see baseline data reported in [23–25]. The behavioral and neural assessments took a total of five hours at each time point and participants were compensated $20 at T1, $150 at T2 and $100 at T3. Data from the neural assessment are in preparation and are not included in this report.

Prior to beginning training, each participant participated in an individually based telephone start-up session with the CWMT coach to become familiarized with the WM training program. Participants were run in cohorts of 15–20 to ensure that the CWMT coach was able to provide each participant with adequate individual attention. Randomization was carried out separately for each cohort by an investigator independent of the research team using a random number generator. Randomization was stratified for medication and sex as males and females with ADHD have shown a differential response to pharmacological treatment and thus they may also show a differential response to cognitive training. During this study, six cohorts were trained within a 12-month period.

### 2.4 Measures

#### 2.4.1 Baseline Measures

1) The Vocabulary and Matrix Reasoning subtests from the Wechsler Abbreviated Scale of Intelligence- Second Edition were administered at T1 as an estimate of general intellectual ability [26], 2) the Math Fluency subtest from The Woodcock Johnson—Third Edition [27] and the Test of Word Reading Efficiency- Second Edition [28] were used to screen basic math and reading skills; 3) the Symptom Assessment 45 was used to assess general psychiatric symptomatology [29], 4) the Kessler-10 [30] was used as a global measure of distress based on questions about anxiety and depressive symptoms and 5) the total score from the ‘GRIT’ scale was used to tap ambition, (e.g. I aim to be the best at what I do), perseverance of effort (e.g. I have overcome setbacks to conquer important challenges), and consistency of interest (e.g. I often set goals but later choose to pursue a different one) in terms of long term goals [31].

#### 2.4.2 Compliance Measures

1) Duration of training: the number of daily sessions participants completed and number of weeks that participants took to complete 25 training sessions. 2) Coach-calls: the number of the five scheduled coach-calls that participants completed. 3) CWMT Training Index Score: this score, which measures the users improvement on selected training tasks, is calculated by subtracting the results of days 2 and 3 of training from the best two days during the training period; the mean index score for individuals 18 to 65 years is 30 (normal range 18–42), with a higher score suggesting good effort during training. 4) Attrition: number of participants who chose to withdraw from the study in each treatment arm prior to completing the required minimum of 20 sessions.

#### 2.4.3 Outcome Measures

Outcome measures were categorized into: 1) Criterion Measures: standardized tests of working memory that closely resemble training activities from CWMT (i.e., untrained tests of WM), and 2) Near-Transfer Measures: measures that tap working memory but do not resemble trained tasks, 3) Far-transfer Measures: measures of transfer to everyday functioning, academic performance, and ADHD symptomology. The majority of the measures were selected based on the results of previous studies [11,18, 32, 33]. Outcome analyses were based on raw scores, which are reported in Tables 2 and 3: standardized scores were used to aid interpretation and are reported in (S2 Table).

**Table 2:** Descriptive Statistics for Criterion, Near-transfer and Far-transfer measures at pre- and post-test (intent to treat).

	Standard-length (n = 32)		Shortened-length (n = 33)		Wait-list Control (n = 32)					
	Pre-test	Post-test	Pre-test	Post-test	Pre-test	Post-test				
Measure	M (sd)	M (sd)	M (sd)	M (sd)	M (sd)	M (sd)	F (1,90)	MSE	P-Value	ŋ^2^
	**Criterion Measures**									
Digit Span: Forwards	10.25 (2.03)	11.32 (2.15)	10.53 (2.19)	11.22 (2.46)	10.03 (1.96)	10.00 (2.08)	4.10	2.82	.020	.084
Digit Span: Backwards	9.09 (2.18)	10.52 (2.10)	9.59 (2.67)	10.53 (2.50)	8.69 (1.96)	8.81 (2.10)	4.67	3.47	.012	.10
Digit Span: Sequencing	9.19 (2.36)	10.55 (2.11)	9.31 (2.56)	9.50 (2.72)	9.44 (2.20)	9.28 (2.23)	4.67	3.64	.012	.10
CANTAB Spatial Span: Forwards	6.88 (1.54)	7.77 (1.02)	7.12 (1.41)	7.70 (1.16)	6.62 (1.43)	6.69 (1.18)	9.16*	.941	.000	.17
CANTAB Spatial Span: Backwards	6.16 (1.35)	7.16 (1.53)	6.63 (1.71)	7.30 (1.53)	6.16 (1.51)	6.29 (1.57)	3.45	1.81	.036	.07
Finger Windows: Forwards	17.63 (3.54)	21.42 (3.31)	20.00 (2.75)	21.00 (3.16)	17.28 (3.93)	18.25 (3.77)	7.84*	8.10	.001	.15
Finger Windows: Backwards	15.13 (3.36)	18.84 (4.16)	17.45 (3.77)	19.39 (3.71)	14.78 (4.42)	16.09 (5.24)	4.18	12.89	.018	.085
	**Near-Transfer Measures**									
CANTAB Spatial Working Memory: Between Errors	19.21 (16.98)	12.10 (15.07)	12.97 (14.77)	12.12 (13.72)	17.47 (14.82)	16.31 (13.62)	2.01	92.84	.14	.04
CANTAB Pattern Recognition Memory	85.68 (10.69)	88.58 (8.47)	86.62 (10.19)	88.76 (9.11)	85.29 (10.69)	89.32 (9.40)	.24	59.00	.79	.005
A Quick Test of Cognitive Speed	8.01 (4.55)	7.57 (3.99)	9.28 (6.65)	7.78 (4.48)	9.48 (4.67)	8.79 (4.50)	.47	8.96	.63	.010
**Far-Transfer Measures**										
Adult ADHD Self-Report Scale	49.00 (9.65)	46.48 (9.89)	48.67 (8.15)	46.63 (9.15)	49.19 (10.78)	47.25 (11.51)	.126	37.34	.88	.003
Cognitive Failures Questionnaire	57.69 (11.62)	54.13 (14.34)	58.00 (13.02)	55.00 (11.21)	58.72 (16.45)	54.05 (16.51)	.49	65.11	.62	.016
Barkley Deficits in Executive Functioning Scale	51.66 (8.61)	49.90 (9.22)	52.38 (11.56)	50.41 (11.00)	53.32 (10.20)	48.13 (11.18)	1.61	41.53	.21	.036
Woodcock Johnson Math Fluency	110.45 (21.88)	115.55(22.25)	112.79 (24.22)	118.33 (23.75)	109.94 (29.16)	114.66 (28.70)	0.59	70.42	.59	.001
Test of Word Reading Efficiency	146.47 (19.40)	148.78(17.03)	148.80 (15.63)	152.83 (14.55)	146.97 (16.25)	153.58 (15.25)	2.08	60.08	.13	.046
	**Non-specific Measures**									
CANTAB Spatial Working Memory: Strategy Score	30.47 (5.90)	27.03 (6.47)	28.27 (6.25)	28.39 (6.79)	29.22 (6.64)	28.59 (6.39)	3.80	18.93	.026	.078
GRIT	2.63 (.52)	2.63 (.69)	2.78 (.54)	2.89 (.55)	2.74 (.56)	2.87 (.60)	1.36	13.46	.26	.029

* Survived Bonferroni correction for multiple comparisons

**Table 3:** Types of post and follow-up treatment effects for Criterion, Near transfer and Far transfer measures.

	Type of treatment effect				
Measure	Dose	High Threshold	Low Threshold	None/ Other	Follow up maintained
**Criterion Measures**					
CANTAB Spatial Span Forwards			✓^a^		
CANTAB Spatial Span Backwards			✓		
Finger Windows Forwards		✓^a^			✓
Finger Windows Backwards			✓		
Digit Span Forwards			✓		
Digit Span Backwards			✓		
Digit Span Sequencing		✓			✓
**Near Transfer Measures**					
CANTAB Pattern Recognition Memory				✓	
CANTAB Working Memory: Between Errors Score				✓	
A Quick Test of Cognitive Speed				✓	
**Far Transfer Measures**					
Adult ADHD Self-Report Scale				✓	
Cognitive Failures Questionnaire				✓	
Berkley Deficits Executive Functioning Scale				✓	
Woodcock Johnson Math Fluency				✓	
Test of Word Reading Efficiency				✓	
**Nonspecific Measures**					
CANTAB Working Memory: Strategy Score		✓			✓
GRIT				✓	

^a^ Withstood Bonferroni correction

Criterion Measures consisted of: 1) Digit Span Forwards, Digit Span Backwards and Digit Span Sequencing from the Wechsler Adult Intelligence Scale—Fourth Addition, were used to assess auditory-verbal working and short-term memory [34], 2) The Spatial Span Forwards and Spatial Span Backwards from The Cambridge Neuropsychological Testing Automated Battery (CANTAB) was used to assess visual-spatial short-term and working memory [35], 3) The Finger Windows Forwards and Finger Windows Backwards subtest from The Wide Range Assessment of Memory and Learning—Second Edition [36], was used as another measure of visual-spatial short-term and working memory.

Near Transfer Measures consisted of: 1) The CANTAB Spatial working memory between errors score was used to assess visual-spatial working memory [35], 2) The CANTAB Pattern Recognition Memory task assessed visual short-term memory [35], 4) A Quick Test of Cognitive Speed was used to measures the speed of naming single dimensions (Color, Form), dual-dimension (ColorForm) and overall processing efficiency [CF–(C + F)] in seconds [37].

Far Transfer Measures consisted of: 1) The Cognitive Failures Questionnaire was used to assess self-reported errors in memory, perception, and motor function when completing everyday tasks; the questionnaire has been found to have good external validity and stability over time [38], 2) The Barkley Deficits in Executive Functioning Scale Short Form, was used to evaluate executive functioning deficits in everyday life activities [39], 3) The Woodcock Johnson-III was used to evaluate math fluency [27], 4) The Test of Word Reading Efficiency-II was used to assess reading fluency [28], 5) The 18-item Adult ADHD Self-Report Scale was used to evaluate current manifestation of ADHD symptoms; this scale is based on the DSM-IV and has high internal consistency and concurrent validity [40].

#### 2.4.4 Non-specific Measures

1) The CANTAB Spatial working memory strategy score was used to assess the ability to adopt an efficient strategy on this working memory task, with a low score indicating good strategy use [35]; and 2) the GRIT [31] was used to assess non-specific training effects on perseverance of effort.

#### 2.4.5 Participant Perspectives

A brief interview was conducted at T2 with each participant in the working memory training groups (but not in the wait-list group) to ascertain their overall experience with the working memory training program, how difficult they found the training, their use of self-reinforcement (as recommended), and any perceived effects from the intervention. Responses were transcribed directly onto the interview form and then coded, thereby yielding both qualitative and quantitative data.

### 2.5 Statistical Analysis

Data were first checked to ascertain the shape of the score distributions using SPSS v.21. A Winsorizing technique described in Tabachnick and Fidell [41] was used to minimize the effect of outliers in each of the following variables: GRIT, Symptom Asessment-45, Cognitive Failure Questionnaire, Barkley Deficits in Executive Functioning Scale, CANTAB Pattern Recognition Memory, CANTAB Spatial Working Memory, CANTAB Spatial Span, Digit span, and Finger Windows Forwards. Outliers occurred randomly across variables and participants in less than 5% of all data points and were not due to any data entry errors.

To analyze the differences from T1 to T2 we used an Intent-to-Treat (ITT) analysis, using the Last Observation Carried Forward for missing data to test for training effects at T2. When all assumptions were valid, an analysis of covariance (ANCOVA) was conducted to optimize control for the variance at baseline. Thus for each outcome measure, T2 datum was the dependent variable, with T1 datum, medication and sex as covariates, and Group (standard-length training, shortened-length training, waitlist control) as the between-subjects variable. Partial eta-squared values (*η* ^2^) were computed to ascertain effect size. According to Vacha-Haase & Thompson [42], *η* ^2^ = .01 corresponds to a small effect, *η* ^2^ = .10 corresponds to a medium effect, and *η* ^2^ = .25 represents a large effect.

To address whether any effects of working memory training were sustained or emerged later, a follow-up analysis was conducted using T3 data. The waitlist control group was not included in this analysis, as this group did not undergo a T3 assessment. The follow-up analyses were conducted on an "as treated" dataset as attrition was higher at T3 (21.5%). In our follow-up analyses, the pre-post results were taken as a basis and ANCOVAs were conducted on the T3 data, covarying out effects at T2. If a strong treatment effect was found at T2 (e.g., a dose or high intensity treatment effect), a significant group effect at T3 would mean that the treatment effects had strengthened or weakened (depending on the pattern of findings), whereas a non-significant effect would indicate that the initial effect at T2 was maintained. Similarly, if a weak or absent treatment effect was found at T2 (e.g., a low intensity or no treatment effect), a significant group effect at T3 would then indicate sleeper effects (e.g., later emerging), whereas non-significant effects would indicate no change from T2. Discrepant outcomes from the main conclusions, after studying the post-hocs, are reported.

Data are presented with and without correction for multiple comparisons. Since a total of 17 tests were conducted for all our outcome measures, the adjusted strict Bonferroni corrected p-value would be .0029 [.05/17] for each of the main outcome measures.

## Results

### 3.1 Baseline Measures

As evident from the mean scaled score partial IQ (Mean = 111.4, SD = 11.89), Digit Span (Mean = 9.13, SD = 2.30), and CANTAB Spatial Span backwards subtest z-scores (Mean = -.25, SD = 1.12), this was a high functioning sample, with approximately 20% having poor auditory-verbal working memory and 13.5% having poor visuospatial working memory (as indicated by scaled scores below the 7th percentile). About 14% (n = 14) scored at or below the 7^th^ percentile (standardized score ≤78) on the Math Fluency Subtest from the Woodcock Johnson-III, with only 2 (0.2%) participants scored at or below the 7th percentile (standardized score ≤78) on the Test of Word Reading Efficiency. The mean T-score of 60.15 for the Symptom Assessment-45 indicated that this sample had above average psychiatric symptomatology with 16% of participants having T-scores above 70 on the Symptom Assessment-45 (i.e., ≥ 2SD above population mean). The mean percentile rank for the Barkley Deficits in Executive Functioning Scale was 94%, indicating that the sample had moderate to severe deficits in executive function. Based on existing norms [30], a mean total score of 37.2 for the Kessler 10 suggesting that high levels of distress were experienced in our sample with 58% experiencing high to very-high levels of psychological distress.

### 3.2 Compliance Measures

1) Duration of training: The standard-length training group, completed the required training sessions in 6.21 weeks (*SD* = 1.45, range: 4.86–10 weeks) with an average of 24.68 sessions completed (SD = 1.11). Participants who did not come in for a T2 assessment (18.75%) completed an average of 4.57 training sessions (*SD* = 5.86, range: 0–12 sessions). In the shortened-length training group, participants completed the required training sessions in 5.65 weeks (*SD* = 1.45, range: 4.43–7 weeks), with an average of 24.84 sessions completed (SD = 0.55). Participants who did not come in for a T2 assessment (6.1%) completed an average of 14.5 training sessions (*SD* = 14.85, range: 4–25 sessions). The data suggest that shortened-length group completed a similar number of sessions (i.e., about 25), but in a shorter time period (5.65 weeks vs 6.21 weeks) than the standard-length group, although the difference was not statistically significant (*t*(54) = 4.37, *p* = .063, *d* = .39).

2) Coach Calls: The majority of participants completed all 5 coach calls; 76% in the standard-length training group, 83.9% in the shortened-length training group, and 90.6% in the waitlist control group, completed all 5 coach-calls. The remaining 9.4% in the waitlist control group did not receive any calls as they dropped out after the initial T1 assessment. There was significant difference in the number of coach calls completed by the groups (*f*(2,97) = 4.3, *p* = .016). The standard-length training group completed significantly fewer coach calls than both the shortened-length (p = .011) and waitlist (p = .015) groups, which did not differ (p = .87).

3) CMWT Training Index score: The majority of participants (75.4%) who completed the training obtained a Training Index Improvement Score within the expected range (i.e., a score of 18 to 41); the average Training Index Score for the standard-length training group was 28.76 (*SD* = 5.43, range: 19–41), and 29.16 (*SD* = 11.12, range: 9–56) for the shortened-length training group. The two CWMT groups did not differ from each other in theTraining Index Improvement Score (*t(54)* = -.80, *p* = .43, *d* = 0.05).

4) Attrition: Only 12 of the 97 participants (12.3%) withdrew from their assigned treatment condition. In the standard-length training group, 25 participants (78.13%) completed their assigned treatment condition and provided T2 data, 31 participants (93.93%) in the shortened-length training group and 30 participants (90.6%) in the waitlist control group. Although more participants in the shortened- than in the standard-length training group appeared to complete the training, the difference was not statistically significant (χ^2^(1) = 3.41, *p* = .065, *r* = 0.25). There were also no significant differences in attrition rates between the 6 cohorts (χ^2^ *(2)* = 2.79, *p* = .25, *r* = 0.18).

### 3.3 Outcome Measures

Descriptive statistics are reported in Table 2, a summary of the findings for post-treatment and follow-up effects are presented in Table 3 shows findings from each category of outcome measure.

### 3.4 Criterion Working Memory Measures

The ANCOVA for the WAIS-IV Digit Span Forwards raw score was significant. Post hoc tests showed that participants in both the standard-length training and shortened-length training group did significantly better at T2 than the waitlist control group (*p* = .009, and .029, respectively), and that there were no significant difference between the two CWMT groups (*p* = .64). Results for the WAIS-IV Digit Span Backwards raw score showed significant differences between the three groups at T2 post-test. Post hoc tests showed that participants in the standard-length training and shortened-length training group did significantly better at T2 than the waitlist control group (*p* = .005, and .019, respectively), and that there were no significant differences between the two training groups (*p* = .65). With respect to the WAIS-IV Digit Span Sequencing raw score, significant differences were found between the groups at T2. Post hoc tests showed that participants in the standard-length training group did significantly better at T2 than both the shortened-length and waitlist groups (*p* = .029, and .004, respectively), which did not differ (*p* = .46).

The ANCOVA for the CANTAB Spatial Span Forwards raw score was significant. Post hoc tests showed that participants in both the standard-length and shortened-length training groups did significantly better than the waitlist control group (*p* = .000, and .001, respectively). There was no significant differences between the standard-length training and shortened-length training groups (*p* = .56). Results for the CANTAB Spatial Span Backwards raw score showed significant differences at T2. Post hoc tests showed that participants in both the standard-length and shortened-length training groups did significantly better than the waitlist control group (*p* = .019, and .033, respectively), but the two training groups did not differ from one another (*p* = .81). The ANCOVA for the Finger Windows Forwards raw score was significant.. Post hoc tests showed that participants in the standard-length training groups did significantly better than those in both the shortened-length and waitlist groups (*p* = .026, and .000, respectively), but the latter two groups did not differ from one another (*p* = .12). Results for the Finger Windows Backwards raw score showed significant differences between the three groups at T2. Post hoc tests showed that participants in the standard-length training group did significantly better than those in the waitlist control group (*p* = .005). There were no significant differences between the shortened-length training and standard-length training groups (*p* = .33), and no differences between the shortened-length training and waitlist control groups (*p* = .07).

After Bonferroni correction (*p* < .0029 [.05/17]), only the CANTAB Spatial Span Forwards raw score (low threshold effect) and the Finger Windows Forwards raw score (High threshold effect) showed statistically significant effects.

### 3.5 Near Transfer Working Memory Measures

With respect to the CANTAB Spatial Working Memory Between Errors score, no significant differences were found between the groups at T2. There were also no differences found at post-test between the groups for the CANTAB Pattern Recognition Memory raw score or the AQT overall processing efficiency score.

### 3.6 Far Transfer Measures

There were no significant differences found between the three groups at post-test for the ASRS total score, CFQ total score, BDEFS total score, TOWRE sum of raw scores (phonemic decoding efficiency and sight word efficiency), and WCJ Math Fluency raw score.

### 3.7 Non-specific measures

The ANCOVA for the CANTAB Spatial Working Memory Strategy Score revealed a significant effect of training. Post hoc tests showed that participants in the standard-length training groups did significantly better than those in both the shortened-length and waitlist control groups (p = .010, and .040, respectively), which did not differ from one another (p = .61). We note that the CANTAB Spatial Working Memory Strategy Score did not survive Bonferroni correction. The ANCOVA for the GRIT total score was not significant (*F*(1,90) = 1.36, MSE = 13.46 *p* = .26, *η* ^*2*^ = .029).

### 3.8 Follow-up Analysis

For the criterion measures, high-intensity threshold effects were found for the Finger Windows Forward and the Digit Span Sequencing. These effects were maintained at T3 since no significant Group effects were found (*p* = .66, and .34, respectively). No Sleeper effects were found for the CANTAB Forwards (*p* = .97) and Backwards (*p* = .89), the Finger Windows Backwards (*p* = .46), and the Digit Span Forwards (*p* = .84), and Backwards (p = .51) analyses. Analyses of remaining five criterion measures, which had yielded a low-intensity threshold effect at T2, did not show a Group effect at T3 or differ from T2. Since no Near-transfer or Far transfer measures showed any treatment effects at T2, our T3 analyses focused on finding Sleeper Effects. Results showed that there were no Sleeper effects for either the Near-transfer analysis for the CANTAB Pattern Recognition Memory (*p* = .30), Between Errors (*p* = .89), and the AQT overall processing efficiency score (*p* = .11)or for the Far transfer measure analyses like the ASRS (*p* = .30), CFQ (*p* = .98), BDEFS (*p* = .18), WCJ Math Fluency (*p* = .36) and the TOWRE (*p* = .98). Finally, the nonspecific treatment effect of the CANTAB Strategy was maintained at T3 (*p* = .70). These results generally show that strong treatment effects that were found were maintained at follow-up and that no Sleeper Effects were found for any of the remaining measures.

### 3.9 Supplemental Analyses

Repeated Measures ANOVAs were also conducted, with Group (3 levels) as a between-subject factor and Time (2) as a within-subject factor, for each of the dependent variables; these results are not reported, as they did not differ from those found with the ANCOVA based on ITT approach. As expected, however, there were main effects for Time for some Criterion measures [Digit Span Backwards (F(1,90) = 4.04, p = .047, u2 = .043); CANTAB Forwards (F(1,91) = 8.05, p = .006, u2 = .081); CANTAB Backwards (1,90 = 11.10, p = .001, u2 = .11); Finger Windows Forwards (F(1,91) = 17.63, p = .000, u2 = .16); and Finger Windows Backwards (F(1,91) = 13.81, p = .000, u2 = .13)]. Also, main effects for Time were significant for some transfer measures [ATQ (F(1,91) = 7.93, p = .006, u2 = .08); ASRS (F(1,90) = 11.06, p = .001, u2 = .11); BDEFS (F(1,88) = 10.33, p = .002, u2 = .11); TOWRE (F(1,88) = 6.08, p = .016, u2 = .07); and the WCJ (F(1,91) = 6.31, p = .014, u2 = .07)], and for one of the non-specific measures [Grit (F(1,90) = 7.85, p = .006, u2 = .08)].

### 3.9 Participant Perspectives

In general, both groups rated the difficulty of training similarly, with more reporting that training was hard in the last week compared to the first week, suggesting participants in both groups were similarly motivated, engaged, and challenged by the adaptive training protocol. More students in the shortened-length training group (61%) reported rewarding themselves after completing a training session, compared to those in the standard-length training group (37%), The most frequently-used reinforcers were a preferred activity, such as playing sports or a videogame (61% of the responses), or food or drinks (39% of responses) Many participants reported that the training experience had been unequivocally good or mostly positive (standard-length training = 53%, shortened-length training = 63%) with very few (a total of 5 participants) indicating that it had been a completely negative experience. Examples of students’ appraisal of CWMT included: “Interesting, sometimes frustrating, but overall a good experience”; “at first frustrating because it was difficult to understand, but then started improving and liked it more”; “Interesting because of the daily challenge but I’m unsure about the long-term effects”; “good, coach calls were helpful, program was easy to use”; “Difficult—a big commitment”; “tedious, games were fun at first but lost their novelty and became a chore once you figured out the system.” Moreover, only some of the participants (standard-length training = 41%, shortened-length training = 57%) thought that CWMT conferred some beneficial effects. Most of those reporting beneficial effects thought that their ability to remember numbers and number sequences (e.g., phone numbers) had improved or simply that they were better at doing the training activities, others reported being better able to focus and concentrate on the task at hand, and a few reported being more organized.

## Conclusions

### 4.1 Discussion

To the best of our knowledge, the current study represents the first randomized controlled clinical trial of CWMT involving participants with ADHD that controls for participants’ motivation, engagement, and expectancy of change. To do so, we compared the outcomes from the standard adaptive and intensive program to those from an active, adaptive but shorter-length version and a contacted waitlist control group. Based on the assumption that WM training needs to be intensive as well as adaptive [9], we predicted either a ‘dose-effect’ pattern of improvements in WM (i.e., standard-length group > shortened-length group > waitlist control), which would provide strong evidence that it was CWMT per se that accounted for improved WM performance, or a ‘high-intensity threshold effect” (i.e., standard-length group > shortened length group = waitlist control), which would support the claimed need for intensive training. In contrast to our hypotheses, the major finding from this study was that the primary pattern of findings conformed to a low-intensity threshold effect (i.e., Standard-length = shortened-length CWMT > untrained wait-list control). Additional findings were that the positive effects on the two standardized tests of WM, which showed a high-intensity threshold effect, appeared to persist for at least 3-months, but we found no evidence of immediate or late-emergent transfer effects to other cognitive functions, ADHD symptoms, or WM performance in daily life. We discuss each of these major findings in more detail below.

A low-intensity threshold effect for CWMT was demonstrated for 5 of the 7 criterion measures, all of which are typically classified as simple span tasks. Our finding of training-related performance changes on all of the untrained WM tasks (criterion tasks) even when using an RCT design that controlled for trainees’ motivation, engagement and expectancy of change, is consistent with findings from recent meta-analysis of CWMT in ADHD populations [15, 19]. Effect sizes for improved WM performance on these tests ranged from .07 to .17 (low-medium to moderately strong). Evidence that the shortened-length group showed similar (or somewhat stronger) compliance with the training protocol, as well as comparable improvements in WM performance, to the standard-length group, indicates that this novel control group did indeed control for motivation, engagement and expectancy of change. However, only 2 of these 7 training-related changes survived more rigorous analysis that corrected for multiple comparisons, namely, performance on two simple visual-spatial span tasks (CANTAB Spatial Span Forwards and the WRAML Finger Windows Forward). These two measures yielded the largest effect sizes for change (0.17, 0.15, respectively), but since they also most closely resemble the CWMT training activities, it is not surprising that trainees’ performance improved on this measure after repeating the adaptive training activities for 25 days.

How are we to account for the unexpected finding that the shortened length group showed comparable gains on the criterion WM tests to those attained by standard-length group? One explanation could be that shorter training sessions are as effective as longer ones for individuals with ADHD who have short-attention spans. This interpretation is suggested by some positive outcomes on WM test performance from shorter-length training (15-min per session, 5 days/week, for 5 weeks) for individuals with short attention spans, such as preschoolers [43, 44]. However, when considering our findings that only 2 of 7 of the criterion measures of WM showed robust changes (i.e., survived Bonferroni correction), a contrasting interpretation is that neither standard-length or shorter-length CWMT programs have any substantial effect on WM performance, at least as assessed in this study in this population. The fact that the most robust training-related changes occurred on two simple span tasks challenges previous claims that CWMT improves performance on complex span tasks as well as simple span tasks [45, 46]. Moreover, the absence of any evidence of dose-dependent effects of training, suggests that the observed changes in WM performance may not be directly attributable to CWMT itself. We speculate whether other factors might have contributed to overall improvement. For instance, positive reinforcement associated with the weekly 30-min coach calls, immediate feedback and rewards based on the accuracy for every trial, self-reinforcement after completing each training session (as self-reported by about 60% of the shortened-length and almost 40% of standard-length group), the need to instigate a daily routine to complete the training, and use of more efficient task-specific strategies (as manifest by CANTAB spatial WM strategy score), might all serve to increase effort and motivation to perform throughout the training period and during post-training assessment. Furthermore, our findings of main effects of Time (pre-post differences) in our supplemental analysis, highlights the importance of using RCT designs: uncontrolled clinical studies are at risk of misattributing pre-post changes to the effects of CWMT.

The second aim of this study was to determine whether training working memory (i.e., a single component of cognition) would induce general transfer to other untrained working memory tasks and cognitive domains. While it is necessary to show evidence of improvement in laboratory-based criterion measures of working memory, this is not sufficient for documenting the efficacy of CWMT. Rather, from both theoretical and clinical perspective the key issue is whether the effects of training also have a positive impact on cognitive and behavioral functioning in everyday life. Contrary to our predictions, not only did we not detect transfer effects on objective measures of cognitive abilities, but also we did not find any on the self-reported measures of executive functioning in everyday life (as measured by the ASRS, BDEFS, CFQ). Since our informants were not blind to the training condition, this finding indicates that these students with ADHD did not manifest a Hawthorne effect or other reporting biases. Unexpectedly, we did find a training-related improvement in task-specific strategy use while performing the CANTAB spatial working memory task. However, improvements in strategy use were not accompanied by improvements in performing that WM task. Our failure to find evidence of transfer effects is not unique to this study: it is consistent with findings from recent meta-analyses [15, 16]. But the key question is why did we, like other researchers fail to find transfer effects? One reason may be our inadequate consideration of measurement issues for capturing transfer effects [47]. Another possible explanation is that the basic premise underlying WM training is flawed. Since there is little or no robust evidence that WM plays a direct causal role in high-level cognition, academic performance, or ADHD symptoms, enhancing WM would not be predicted to have strong or direct effects on these abilities.

Our third aim was to determine whether training related improvements were sustained after ending training. Notwithstanding the limitations of our design to provide a robust test of sustained effects, we did find evidence for sustained effects on 3 measures: two standardized tests of WM (notably, those that yielded the largest effect sizes for immediate training-related changes) and one non-specific measure of task-specific strategy use. Notably, all 3 of these outcome measured showed a high-intensity threshold pattern of effects. However, that participants undergoing standard-length intensive CWMT improved in task-specific strategy use, is consistent with a recent finding [48] that participants undergoing CWMT reported more frequent use of strategies while undertaking the training. Thus, this finding raises the question of whether intensive training of a single component of cognition (i.e., WM) may induce the development of task-specific strategies in an attempt to alleviate cognitive load [49], rather than directly improving WM per se.

### 4.2 Limitations

While the design of this study was quite strong due to the utilization of an active, adaptive shortened-length training group as well as a contacted wait-list group, there were also limitations. First, our sample of post-secondary students with ADHD was relatively high functioning and had relatively intact working memory skills—as indicated on clinical standardized tests of working memory compared to other subgroups of ADHD. Thus, there may not have been much room for improvement with regards to their WM skills and so our findings cannot be generalized to the larger ADHD population, even though WM impairments are not universal in ADHD. However, CMWT is claimed to improve working memory in healthy population with adequate working memory and there are studies that have shown this [50–52]. Also, the measures implemented to detect transfer effects in this study may not have been sufficiently sensitive to detect change over a period of a few weeks. We used measures commonly used in previous CWMT studies, but acknowledge that more precise and temporally-contingent measures of attention during task performance may be more sensitive to transfer effects [50]. Nonetheless, we did include self-report measures of executive function (BDEFS) and Cognitive Failures (CFQ) and neither revealed significant transfer effects. In addition, our ability to detect sustained effects of training at the 3-month follow-up assessment was limited by the lack of follow-up data for the waitlist control group. We were required to offer CWMT training to waitlist control participants while they were still attending post-secondary education, and so we were not able to put their training on hold for a 3-month period. Finally, it is also possible that a follow-up period of 3 months is not sufficient to detect later-emerging or ‘sleeper’ effects, as has been reported [53].

### 4.3 Conclusion

The key question concerning the effectiveness of CWMT is not simply whether it increases working memory on trained tasks, but also whether it enhances working memory in everyday life. We conclude that standard CWMT as it is currently offered, is not an effective approach for improving working memory in post-secondary students with ADHD, as measured by standardized WM tests or in everyday life. Moreover, the overall pattern of our findings raises questions about the specificity of training effects on working memory. Though these results may seem discouraging for the field, we commend the openness of CogMed to independent scientific scrutiny. This allows the field to progress and adapt treatment in ways that may be more beneficial.

## Supporting Information

S1 Table CogMed Tasks. (DOCX)

S2 Table Standardized Scores. (DOCX)

S1 CONSORT Checklist CONSORT checklist. (DOC)

S1 Protocol Ethics Protocol Submission. (DOCX)
