# Supplementary material for: Working Memory Training in Post-Secondary Students with ADHD: A Randomized Controlled Study
Source: PLoS One. 2015 Sep 23;10(9):e0137173. doi: 10.1371/journal.pone.0137173 (PMC4580470; doi:10.1371/journal.pone.0137173)
Supplement: S1 Table — (DOCX) [file pone.0137173.s001.docx]

**Table 2: Description of WM Tasks in the Cogmed WM Training Program**

| **Task** | **Description** |
| --- | --- |
| 1. Reproducing a light sequence in a visuo-spatial grid | Lamps arranged in a 4X4 grid are displayed. Participants watch several lamps light up and then reproduce the same sequence. |
| 2. Reproducing a light sequence in a rotated grid | A rotating version of the grid task described above. After the sequence of lamps lights up, the panel rotates 90 degrees clockwise and participants reproduce the sequence in the panel’s new position. |
| 3. Repeating numbers in reverse order | A keyboard with numbers is displayed and then numbers are read aloud. Participants respond by repeating the digits in reverse order. |
| 4. Repeating numbers in reverse order with an invisible keyboard | Similar to the task described above, but numbers are not visible on the screen until participants are required to respond. |
| 5. Identifying letter positions in a sequence | Letters are read aloud, one at a time. Participants have to remember the letters in the order in which they are read. A row of lights becomes visible and a flashing light cues the participant to respond. For example, if light number 3 lights up, then participants report the 3^rd^ letter they heard. |
| 6. Identifying letter sequences | A sequence of letters is read aloud. Then, the participant is presented with three letters and must select the one that was presented. |
| 7. Reproducing a light sequence in a rotating circle | A set of lamps is arranged in a rotating circle. Participants watch several lights light up and then reproduce the sequence, even though the lamps are constantly shifting position. |
| 8. Reproducing a light sequence | A number of moving circles appear on screen and participants must reproduce the order in which they appeared. |
| 9. Reproducing a sequence of moving shapes | A number of moving shapes light up and participants must reproduce the sequence in which they lit up. |
| 10. Reproducing a light sequence in a 3D visuo-spatial grid | Lights are arranged in a ‘3D room’ with 20 segments. Participants watch several lights go on and then reproduce the sequence. |
| 11. Reproducing a light sequence in a 3D visuo-spatial cube | Lights are positioned in a 3D cube with 12 segments. Participants watch several lights go on and then reproduce the sequence. |
| 12. Reproducing a sequence of numbers on a visual grid | A 4X4 grid with 16 latches is shown. A sequence of latches is opened displaying a set of numbers. Participants sort the numbers by clicking on the latch that contained the numbers in numerical order. |
