# Supplementary material for: Working Memory Training in Post-Secondary Students with ADHD: A Randomized Controlled Study
Source: PLoS One. 2015 Sep 23;10(9):e0137173. doi: 10.1371/journal.pone.0137173 (PMC4580470; doi:10.1371/journal.pone.0137173)
Supplement: S2 Table — (DOCX) [file pone.0137173.s002.docx]

Supplementary Table 1

*Descriptive Statistics of Standardized scores for Criterion, Near-transfer and Far-transfer measures at the pre- and post-test*

| Measure | Standard-length Training Group (n=32) | | | | | Shortened-length Training Group (n=33) | | | | | | Wait-list Control Group  (n=32) | | | | |
| --- | --- | --- | --- | --- | --- | --- | --- | --- | --- | --- | --- | --- | --- | --- | --- | --- |
|  | Pre-test | | | Post-test | | Pre-test | | | Post-test | | | Pre-test | | Post-test | | |
|  | M | SD | M | | SD | | M | SD | M | SD | M | | SD | | M | SD |
|  | | | | | | | | | | | | | | | | |
| **Criterion Measures** | | | | | | | | | | | | | | | | |
| WAIS Digit Span (SS) | 8.81 | 3.02 | 11.68 | | 3.14 | | 9.38 | 3.12 | 10.52 | 3.53 | 8.56 | | 2.51 | | 9.41 | 4.82 |
| CANTAB Spatial Span: Forwards (z) | .09 | 1.16 | .80 | | .75 | | .39 | 1.18 | .91 | .96 | -.10 | | 1.11 | | -.00 | .96 |
| CANTAB Spatial Span: Backwards (z) | -.39 | .94 | .32 | | 1.12 | | .08 | 1.27 | .61 | 1.16 | -.45 | | 1.09 | | -.26 | 1.22 |
| WRAML Finger Windows: Forwards (SS) | 9.47 | 4.20 | 13.16 | | 4.08 | | 11.42 | 3.07 | 12.76 | 3.57 | 9.16 | | 3.57 | | 10.00 | 3.71 |
| **Near-Transfer Measures** | | | | | | | | | | | | | | | | |
| CANTAB Spatial Working Memory: Between Errors Score (z) | -.32 | 1.27 | .22 | | .90 | | .16 | 1.10 | .26 | .83 | -.30 | | 1.19 | | -.13 | .96 |
| CANTAB Pattern Recognition Memory (z) | -.07 | .93 | .09 | | .92 | | -.03 | .90 | .07 | .91 | -.11 | | .93 | | .25 | .79 |
| **Far-Transfer Measures** | | | | | | | | | | | | | | | | |
| CFQ (% > 54.7) | 61.5% | -- | 43.3% | | -- | | 68.0% | -- | 58.6% | -- | 56.0% | | -- | | 37.0% | -- |
| BDEFS (% rank) | 94.77 | 7.19 | 92.39 | | 9.33 | | 92.13 | 12.00 | 90.91 | 14.37 | 94.65 | | 7.77 | | 87.87 | 15.64 |
| WCJ Math Fluency (SS) | 93.32 | 10.21 | 96.43 | | 10.25 | | 92.61 | 11.59 | 96.03 | 11.11 | 91.97 | | 13.31 | | 94.19 | 13.22 |
| TOWRE: Sum of Sight Word Reading and Phonemic Decoding Efficiency Subscales (SS) | 105.69 | 13.07 | 107.23 | | 11.69 | | 107.03 | 10.67 | 110.82 | 11.92 | 105.56 | | 10.56 | | 109.81 | 10.63 |
| **Non-specific Measures** | | | | | | | | | | | | | | | | |
| CANTAB Spatial Working Memory: Strategy Score (z) | -.11 | 1.08 | .50 | | 1.11 | | .34 | 1.12 | .31 | 1.21 | .05 | | 1.27 | | .24 | 1.12 |
