# Supplementary material for: Working Memory Training in Post-Secondary Students with ADHD: A Randomized Controlled Study
Source: PLoS One. 2015 Sep 23;10(9):e0137173. doi: 10.1371/journal.pone.0137173 (PMC4580470; doi:10.1371/journal.pone.0137173)
Supplement: S1 Protocol — (DOCX) [file pone.0137173.s004.docx]

UNIVERSITY OF TORONTO

Office Use Only

Protocol Number:

Office of the Vice-President, Research

Office of Research Ethics


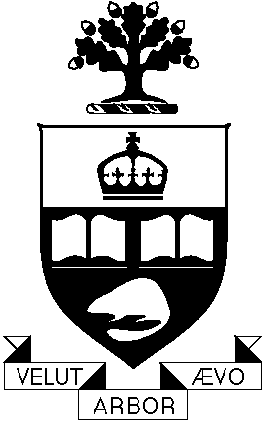


# ETHICS REVIEW PROTOCOL SUBMISSION FORM FOR

# SUPERVISED AND SPONSORED RESEARCHERS

(For use by graduate students, post-docs and visiting professors and researchers)

| **SECTION A – GENERAL INFORMATION** |
| --- |

1. **TITLE OF RESEARCH PROJECT**

| Working Memory Training in College Students with ADHD/LD |
| --- |

**2. INVESTIGATOR INFORMATION**

**Investigator:**

| Title: Mr. | Name: **Steven Woltering** | | |
| --- | --- | --- | --- |
| Department: Human Development and Applied Psychology | | | |
| Mailing address: 252 Bloor St. W., 9^th^ floor M5S-1V6 | | | |
| Phone: (416) 978 1003 | | Fax: | Email: steven.woltering@ utoronto.ca |

**Level of Project**

| Faculty Research |
| --- |
| Post-Doctoral Research X |
| Student Research: Doctoral  Masters  Student Number |

**Faculty Supervisor/Sponsor:**

| Title: Dr. | Name: Rosemary Tannock | | |
| --- | --- | --- | --- |
| Department: Human Development and Applied Psychology | | | |
| Mailing address: 252 Bloor St W, Rm. 9-288 M5S-1V6 | | | |
| Phone: (416) 978-0924 | | Fax: (416) 926-4708 | Email: rosemary.tannock@utoronto.ca |

**Co-Investigators:**

Are co-investigators involved? Yes  No

| Title: Assoc Prof | Name: Maggie Toplak | | |
| --- | --- | --- | --- |
| Department: York University, Department of Psychology | | | |
| Mailing address: 4700 Keele St. Toronto, ON M3J 1P3 | | | |
| Phone: 416-736-2100, ext. 33710 | | Fax: | Email: mtoplak@yorku.ca |

| Title: Dr. | Name: Umesh Jain | | |
| --- | --- | --- | --- |
| Department: Child and Adolescent Psychiatry | | | |
| Mailing address: Centre for Addiction & Mental Health | | | |
| Phone: | | Fax: | Email:   [umesh_jain@camh.net](mailto:umesh_jain@camh.net) |

*Please append additional pages if necessary.*

1. **UNIVERSITY OF TORONTO RESEARCH ETHICS BOARD**

Health Sciences  Education  Social Science & Humanities

Please consult <http://www.research.utoronto.ca/ethics/eh_rebs.html> to determine which Research Ethics Board your proposal should be submitted to.

1. **LOCATION(S) WHERE THE RESEARCH WILL BE CONDUCTED:**

If the research is to be conducted at a site requiring administrative approval/consent (e.g. in a school), please include all draft administrative consent letters. It is the responsibility of the researcher to determine what other means of approval are required, and to obtain approval prior to starting the project.

University of Toronto

Hospital        specify site(s)

School board or community agency        specify site(s)

Community within the GTA        specify site(s)

International        specify site(s)

Other        specify site(s)

**The University of Toronto has recently reached an agreement with the University-Affiliated Teaching Hospitals, regarding ethics review of hospital-based research. Based on this agreement, certain hospital-based research is now exempt from ethics review at the University of Toronto. If your research is based at a University-Affiliated Teaching Hospital please consult the following document to determine whether or not your research requires review at the University of Toronto** <http://www.research.utoronto.ca/ethics/eh_where_tahsn.html>**.**

**5. OTHER RESEARCH ETHICS BOARD APPROVAL(S)**

(a) Does the research involve another institution or site? Yes X No

(b) Has any other REB approved this project? Yes X No

If **Yes** please provide a copy of the approval letter upon submission of this application.

If **No**, will any other REB be asked for approval?

Yes        (please specify which REB) No

**6. FUNDING OF THE PROJECT**

(a) Please check one:

| Funded | Agency: Canadian Institutes of Health Research | Fund #: **245899** |
| --- | --- | --- |
|  | Agency: Canada Research Chairs Program (Salary support for Dr Tannock) | Fund #: N/A |
| Applied for funding | Agency: | Submission date: |
|  | Agency: | Submission date: |
| Unfunded | | |

**If one protocol is to cover more than one grant, please include all fund numbers.**

(b) If waiting for funding, do you wish to postdate ethics approval to the release of funds?

Yes  No

(c) For funded research, will more than one protocol be submitted to cover all research funded by the respective grant? Yes  No X

If **Yes**, this is #

**7. CONTRACTS**

Is there a funding or non-funded agreement associated with the research?

Yes  No

If **Yes**, please include 3 copies upon submission of this application. The contract between Pearson/Cogmed and University of Toronto is in process.

**8. PROJECT START AND END DATES**

Estimated start date for this project: Jan 2012 (for the start of amended protocol)

Estimated completion date for this project: August 2014

**9. SCHOLARLY REVIEW**

Please check one:

The research has been approved by a thesis committee, or equivalent (required for thesis research)

X The research has undergone scholarly review prior to this submission for ethical review

**Scientific peer review by CIHR**

The research will undergo scholarly review prior to funding

      (specify review committee)

The research will not undergo scholarly review apart from this ethics review

**10. CONFLICTS OF INTEREST**

(a) Will the researcher(s), members of the research team, and/or their partners or immediate family members:

(i) Receive any personal benefits (e.g. financial benefit such as remuneration, intellectual property rights, rights of employment, consultancies, board membership, share ownership, stock options, etc.) as a result of or in connection to this study? Yes  No

(ii) If **Yes**, please describe the benefits below. (Do not include conference and travel expense coverage, or other benefits which are standard to the conduct of research.)

NO, except that Cogmed-Pearson provide the research software licences without cost for this research study

(b) Describe any restrictions regarding access to or disclosure of information (during or at the end of the study) that has been placed on the investigator(s). This includes controls placed by sponsor, advisory or steering committee.

None

(c) Where relevant, please explain any pre-existing relationship between the researcher(s) and the researched (e.g. instructor-student; manager-employee; minister-congregant).

N/A

| **SECTION B – SUMMARY OF THE PROPOSED RESEARCH** |
| --- |

**Please include a list of appendices for all additional materials submitted.**

**11. RATIONALE**

Attention-Deficit/Hyperactivity Disorder (ADHD) and Learning Disability (LD) are among the most common neurobehavioural disorders, with prevalence rates estimated at about 5% to 9% ([1](#_ENREF_1), [2](#_ENREF_2)). Approximately 4% of youth in the United States have a comorbid diagnosis of ADHD and a Learning Disability (ADHD/LD) ([3](#_ENREF_3)). Research has shown ADHD/LD symptoms persist into young adulthood, a time when many are enrolled in post-secondary education. Students with ADHD/LD at the post-secondary education level constitute an emergent subgroup of the ADHD and LD populations that have received far less attention in the literature compared to children, adolescents and employed adults. The actual percentage of college students with ADHD/LD is unknown; however, some estimates suggest that 2-8% of students attending post secondary education have ADHD, LD or both (3). Youth with the additive problems of both disorders are at high risk for academic failure, and poor psychosocial and occupational outcomes in adulthood ([3](#_ENREF_3)). The “multiple deficit model” suggests that there is a common genetic and neuropsychological underpinning to these disorders ([3](#_ENREF_3)). For example, ADHD and LD share similar features, such as core deficits in processing speed and working memory ([4](#_ENREF_4), [5](#_ENREF_5)). ADHD and LD are each associated with several cognitive difficulties including poor working memory (WM) and processing speed ([4](#_ENREF_4), [5](#_ENREF_5)). WM is a “mental workspace” that provides temporary storage and manipulation of information and is closely related to *g*, a proposed measure of general cognitive ability ([6](#_ENREF_6)). In addition, WM has also been found to predict academic achievement ([7-9](#_ENREF_7)). One major problem is that current intervention approaches for ADHD/LD do not target the underlying cognitive deficits fundamental to these diagnoses. Thus, interventions that address underlying cognitive difficulties, such as WM, are a promising avenue of additional treatment for youth with combined ADHD/LD.

WM capacity has generally been thought to be a fixed trait, but recent studies have suggested that it can be improved by intensive and adaptive computerized training ([10](#_ENREF_10)). This intervention approach has been evaluated in children and adolescents with ADHD, older adults, and adult stroke patients and shown promising results ([11-14](#_ENREF_11)). Subjects not only improved on the trained WM tasks, but some of the studies suggest that improvements may generalize to non-trained WM activities, complex reasoning tasks, academic functioning, and behavioral symptoms of ADHD or working memory failure ([11](#_ENREF_11), [15](#_ENREF_15)). Moreover, brain imaging studies have provided converging evidence of training-related improvements in working memory: specifically increased activation has been found in cortical regions implicated in working memory ([14](#_ENREF_14), [16](#_ENREF_16), [17](#_ENREF_17)). However, no studies to date have investigated whether WM capacity can be improved in a population of young adults with ADHD/LD enrolled in post-secondary education programs. Nor have the results been replicated or elaborated upon using different imaging methodologies, like EEG (Electroencephalography), which is able to capture the millisecond time parameters of cognitive processing and so may provide new insights into the neural mechanism of WM and effects of WM training.

The overall objective of the current study is to determine the effectiveness of WM training, as administered by community-based psychologists licensed to provide this training, for college students with ADHD/LD. Specific objectives are to determine whether; i) high-intensity computerized WM training enhances WM capacity in college students with ADHD/LD, using behavioural as well as neuroimaging indices of change; ii) lower-intensity training would also result in improvements in WM; iii) WM training normalizes WM, as compared to typically developing peers (i.e., a healthy comparison group of college students); iv) improvements in WM will generalize to secondary outcome tasks, such as inhibitory control and planning; v) WM training will also ameliorate ADHD symptoms of inattention and hyperactivity; and vi) improvements will be sustained for at least a few months after completing the training.

**12. METHODS**

*Intervention*

The Cogmed Working Memory Training Program ([www.cogmed.com](http://www.cogmed.com)) will be used as the experimental program because of preliminary evidence indicating its effectiveness in enhancing WM ([10](#_ENREF_10), [11](#_ENREF_11), [13](#_ENREF_13), [14](#_ENREF_14)). Training is implemented with a software program “Cogmed QM” © from Pearson/Cogmed. It includes a set of auditory verbal and visual-spatial WM tasks presented via computer. All tasks involve: maintenance of simultaneous mental representations of multiple stimuli, unique sequencing of stimulus order in each trial and progressive adaptation of difficulty level as a function of individual performance.

There are a total of 13 different WM training exercises that include both auditory verbal and visual spatial modalities. Participants complete 8 of the 13 tasks every day, 15 trials of each task. An example of a visual spatial task is “3D grid”. In this task, there is a 3D box that is comprised of smaller boxes. The boxes light up in sequence and the participant must click on the boxes that lit up in the correct order. Task demands intensify as the number of boxes that light up increases. An example of an auditory verbal task is “Hidden”. In the task, a voice recites a series of numbers then a grid appears on the screen with numbers 1 through 9 and participants must click on those numbers they heard aloud, but in reverse order. Task demands intensify as the number of numbers increases.

The traditional training program is intensive, requiring approximately 30 min/day, 5 days a week for 5-6 weeks. Participants are required to complete 90 WM trials on each training day. Training plans are individualized and are modified according to performance, but the typical plan includes 13 tasks with 15 trials of 8 tasks each day. Responses are automatically logged to an electronic file, which is uploaded via the internet to servers at JVS to verify compliance.

*Study Design*

The study uses a randomized controlled design in which participants are randomly assigned to one of three groups: standard-length version of high-intensity WM training, a novel shorter version of the high-intensity WM training, or a wait-list control group. The standard-length WM training group will undergo 25 training sessions for 30 min/day, 5 days a week for 5 weeks and receive weekly phone calls from a cogmed coach to review progress and adjust the training as needed; the group assigned to the shorter version of the high-intensity training will also undergo 25 training sessions but for just 15 min/day, 5 days per week for 5 weeks and also receive the same type of weekly phone calls from a cogmed coach; and the wait-list group will not undergo any WM training during the 5 week period but will receive weekly phone calls from a member of the research team to review progress and advice on time management, organization, and mnemonic strategies. All groups will undergo pre testing, testing three weeks following completion of training and again at three-month follow-up. The wait-list group will be offered the cogmed training after the follow-up assessment. We will also recruit a group of healthy age- and gender-matched peers who will be assessed once and will not undergo WM training; this comparison group allows us to determine initial group differences in behavioral and neural indices of WM and whether WM training normalizes these indices in the ADHD groups.

*Diagnostic screening measures* (Baseline assessment only for AHD/LD and healthy controls).

All participants must have received a prior diagnosis of ADHD/LD to register with the college/university Student Disability Services. Since there is no standardized assessment used by Student Disability Services, we will administer a brief interview and standardized rating scales and screening tests to quantify the current symptoms of ADHD, and other clinical problems such as mood and stress levels.

*Adult ADHD Self-Report Scale* (ASRS v1.1) is a reliable and valid scale for evaluating ADHD in adults ([20](#_ENREF_20), [21](#_ENREF_21)). The ASRS v1.1 is an instrument consisting of eighteen questions based on the criteria used for diagnosing ADHD in adults using DSM-IV-TR criteria. Questions focus on how often ADHD symptoms occur (0=never, 1=rarely, 2=sometimes, 3=often, 4=very often). Scores for each item are added to calculate a total score.

*Kessler-10 (K10+).* The interviewer administered K10+ is a widely used psychological screening tool that aims to collect information on the individual’s current symptoms and to establish a productive dialogue. The 10-items yield a global measure of distress based on non-invasive questions about anxiety and depressive symptoms that an individual has experienced in the most recent 4-week period. Answers are multiple choice. <http://www.hcp.med.harvard.edu/ncs/ftpdir/k6/IWER%20K10.pdf>

*Symptom Assessment-45 (SA-45)* The SA–45 is a brief screen for various mental health symptoms, based on the well-validate longer version (SCL–90–R). It is often used as a screening tool, to help in diagnosis, to develop treatment plans, and to measure outcomes. Its items cover symptoms of obsessive compulsivity, psychoticism, somaticization, sensitivity to others, hostility to others, paranoid ideation, phobias, and depression. It is used in this study to allow us to describe the sample and to provide continuous measures of other types of psychopathology that might contribute to cognitive function

*Woodcock Johnson-III Tests of Achievement* ([18](#_ENREF_18)). Participants will complete three subtests: Math Fluency and Reading Fluency tasks determine automaticity of identifying words and recalling math facts; the Applied Problems subtest assesses mathematical reasoning ability. Scores below 16^th^ percentile will be used to support an LD diagnosis.

*Wechsler Abbreviated Scale of Intelligence (WASI).* This is a quick and reliable measure of intelligence ([19](#_ENREF_19)). The WASI consists of four subtests: Vocabulary, Similarities, Block Design, and Matrix Reasoning. In the interest of time, we will only administer the 2-subtests form, which will yield an estimate of general intellectual ability.

*Colour Perception Screening Tests*. Several of our key measures use coloured stimuli. Thus we will screen for color perception using two brief clinical measures: The *Farnsworth-Munsel 100 Hue Test* ([22](#_ENREF_22)) and the *Mollon-Refflin Minimalist Colour Vision Test (*[*23*](#_ENREF_23)*),* which screen for red-green and blue-yellow perception problems.

***Behavioral Outcome Measures:***

Outcome measures were categorized into (1) Compliance measures’ (number of training sessions and compliance with the required training intensity as quantified by the Cogmed Improvement Index score); (2) ‘Criterion measures’ (closely resembled tasks trained in WM program); (3) ‘Near transfer” measures (indices of other cognitive functions or measures of WM that differed from the trained tasks); and (4) ‘Far transfer’ measures (indices of academic achievement and self-report questionnaires). These measures were selected based on the results of previous studies ([24](#_ENREF_24), [25](#_ENREF_25)).

***Compliance measures***

Three measures will be used: i) Number of training sessions completed; ii) Mean proportion actively engaged in training while logged in, as recorded by the software program; and iii) the Cogmed Improvement Index score, which is computed automatically from two training indices (Start Index, Max Index) and provided for every user. The Start Index is calculated with the results from days 2 and 3, and the Max Index is calculated with the results from the two best days during the training period. The Improvement Index is calculated by subtracting the Start Index from the Max Index. The mean Improvement Index for individuals aged 18 to 65 years is 29 (normal range 15-41). Higher Index scores signify good compliance and effort with the training.

***Criterion Measures*** (similar to the trained WM activities)

i) Auditory-Verbal WM: Two measures are to be used. The Digit Span subtest from the *Wechsler Adult Intelligence Scale- Fourth Edition* (WAIS-IV) ([26](#_ENREF_26)) and the *‘Add-3’* WM test that requires participants to add ‘3’ to each digit in a set of 4 digits presented at a constant rate (e.g., the response to the set ‘3 1 5 4’ should be ‘6 4 8 7’ ([27](#_ENREF_27))

ii) Visual-Spatial WM: Two measures are used. The *Cambridge Neuropsychological Testing Automated Battery* *Spatial Span task* was used to assess visual-spatial WM (CANTAB) ([28](#_ENREF_28)) and the ‘*Finger-Windows’* subtest from the *Wide Range Assessment of Memory and Learning* ([29](#_ENREF_29)). The CANTAB task presents a set of white squares on a screen, which momentarily change colour in a variable sequence. The participant must then touch the boxes in the same order that they changed colour on the screen. Task demands intensify, as the number of boxes is increased from two to nine. However, if the participant makes an error, the next trial remains at the same difficulty level. Spatial span scores range from 0-9, with the score representing the highest level at which the participant reproduces at least one correct sequence.

***Near-Transfer Measures*** (other cognitive measures that differ from trained activities)

Four measures are used. The *CANTAB Spatial Working Memory task* is to be used to differentiate and quantify WM memory and strategy skills. Based on the self-ordered pointing task ([30](#_ENREF_30)) , it differs from WM span tasks because it is not affected by varying levels of dopamine in the dorsolateral prefrontal cortex ([31](#_ENREF_31)). Participants are required to update information about spatial locations in working memory: they must search boxes to find the hidden token, but must not return to the same box where a token has already been found. There are four trials for each of three load conditions (four, six and eight boxes). Performance was measured according to the number of errors made. At the end of the task, subjects received a strategy score: a low score indicates efficient strategy use, whereas high scores reflect poor strategy use.

*CANTAB Stockings of Cambridge (planning)*

The subject is shown two displays, each containing three balls, with each set arranged in different “stockings” hanging in three pockets. The subject must move the balls in the lower display to copy the pattern in the upper display. This involves working out an optimal set of moves (the fewest moves possible) and then executing them by moving one ball at a time.

*A Quick Test of Cognitive Speed* (AQT) consists of three rapid naming tasks (colour, form, color-form) and measures processing speed, cognitive set shifting between visual dimensions and semantic fields, and activation of working memory for processing and monitoring ([26-29](#_ENREF_26)).

*Behavioural Assessment of the Dysexecutive Syndrome (BADS).* This validated assessment battery assesses executive function in everyday life that consists of 6 independent tasks; it is sensitive to mild cognitive impairment in adults as well as more severe impairment ([32](#_ENREF_32), [33](#_ENREF_33)). We will use two of the tests: Rule Shift Cards and Zoo Map.

***Far-Transfer Measures***

*ASRS v.1.1* . This scale will also be used as an outcome measure at post-test and follow-up to ascertain any change in ADHD symptoms ([20](#_ENREF_20), [21](#_ENREF_21)).

*Cognitive Failures Questionnaire (CFQ).* This tool measures self-reported failures in perception, memory, and motor function in every day life. This 25-item scale has good external validity ([34](#_ENREF_34)). Questions require subjects to rank how often these mistakes occur (0=never, 1=very rarely, 2=occasionally, 3=quite often, 4=very often). Scores for each item are added to calculate a total score.

*Barkley Deficits in Executive Functioning Scale (BDEFS).* This questionnaire tool will be used to evaluate dimensions of adult executive functioning and working memory in daily life ([35](#_ENREF_35), [36](#_ENREF_36)). We will use the short form.

***Neuroimaging Outcome Measures:***

Three well-validated measures of working memory and related processes will be administered in the EEG lab at OISE, adjacent to the cognitive lab where the behavioral measures are administered.

Go-nogo task (response inhibition)

The subject is shown sequences of letters and instructed to press a button whenever a letter shows up on the screen, but to withhold a response whenever a letter occurs twice in a row. This task will take 8 minutes and is adapted from Garavan, Ross, & Stein (1999).

Delayed Working memory task (working memory)

The subject is shown sequences of either 2 (low load) or 4 (high load) shapes. After a delay period of two seconds, a probe appears and the participant needs to respond by pressing a button whether the probe stimulus was part of the sequence shown before or not. This task will take 16 minutes and is adapted from Haenschell et al., (2007).

Selective Working memory task (selective attention + working memory)

The subject is shown an array of small colored squares to different parts of their visual field. This memory array will consist of either 2 (low load), 4 (high load), or 2 (low load + 2 ‘distractor’ circle) items. After a delay, a test-array is shown and the participant is to respond whether the test-array matched the memory array. This task will take 24 minutes and is adapted from Vogel, McCollough, & Machizawa (2005).

*Imaging equipment*

EEG (Electroencephalography) will be recorded using a 128-channel Geodesic Sensor Net (Tucker, 1993). The net consists out of sponges which contain sensors which allow the measurement of brain activity on the surface of the head.

The basic approach to tests the effects of the training program will be to use repeated measures ANOVA. Separate analyses will be conducted for the primary, secondary and tertiary outcome measures.

For the imaging, raw EEG will be sampled at 500 Hz, using EGI software (Electrical Geodesic, Inc., Eugene, OR) and further processed using EGI software (Electrical Geodesics, Inc., Eugene, OR) after which ERP (Event Related Potential: averaged brainwaves) measures will be created and analyzed in SPSS. Repeated measures ANOVA’s will be used to compare levels of brain activity in various regions before and after training.

**13. PARTICIPANTS OR DATA SUBJECTS**

The sample will include college students with ADHD and/or Learning Disabilities attending the University of Toronto, York University, Humber, Ryerson, or George Brown. The estimated sample size is 160 participants (40 in experimental, 40 in low intensity, 40 in waitlist, and 40 normal healthy controls). Participants will be of no relationship to the investigators. Those who participate in the study must be enrolled as full time students. All participants must have received a previous diagnosis of ADHD and/or a Learning Disability. Exclusionary criteria will include uncorrected sensory impairment, evidence of a neurological dysfunction or a history of psychosis. Personal information, and checks on eligibility, will be obtained directly from the participants during the intake procedures which are appended to this proposal. Ethics approval will be sought for institutions other than the University of Toronto in accordance with those institutions’ policies and procedures.

**14. EXPERIENCE**

The faculty investigator has extensive research working with this population and is considered an international expert in the field of ADHD/LD. Currently, she holds a Canada Research Chair (Tier 1) in Special Education at OISE/University of Toronto, where she is a Professor in Special Education and in Psychiatry; she is also a senior scientist in the Neurosciences and Mental Health Research program at the Hospital for Sick Children. She supervised the doctoral student, Rachel Gropper, who was listed as Primary Investigator on the original protocol for this study, approved by the university Research Ethics Board.

Mr Steven Woltering, who has completed his doctoral research and expects to defend in his thesis in a couple of months time, will then become Dr Tannock’s Post Doctoral Student. He will supervise the neuroimaging component of the project and assist Dr Tannock in supervising the behavioral components of the project. He has obtained various international EEG certifications from basic and advanced EGI trainings (2006, 2007) as well as bootcamps (2008) from renowned ERP researchers, such as Dr. Steven Luck. These, in combination with more than 3 years of experience with the EEG system at the University of Toronto demonstrate sufficient expertise with the various procedures of conducting EEG research. Mr Woltering has played a major role in designing the modified protocol and in writing the CIHR grant that supports this ongoing project.

**15. RECRUITMENT**

Participants will be recruited through the Accessibility Services from several universities and colleges in and around Toronto, such as the University of Toronto, York University, Ryerson, George Brown, and Humber. Students will be notified about an information session will be held on campus describing the Cogmed Working Memory Training Program as well as the proposed research study. At that time, students will be given the opportunity to sign-up to participate if they are interested.

**16. COMPENSATION**

(a) Will participants receive compensation for participation?

**Financial** Yes  No

In-kind Yes  No

Other Yes  No

(b) If **Yes**, please provide details.

Participants will receive $40 for the first visit, $80 for the second visit, and $80 for the last, follow-up visit. The comparison group will receive $60 for their visit.

(c) Where there is a withdrawal clause in the research procedure, if participants choose to withdraw, how will you deal with compensation?

Participants will be paid for their presence at the testing lab, even if they withdraw during that testing session.

| **SECTION C –DESCRIPTION OF THE RISKS AND BENEFITS OF THE PROPOSED RESEARCH** |
| --- |

**17. POSSIBLE RISKS**

1. Indicate if the participants as individuals or as part of an identifiable group or community might experience any of the following risks by being part of this research project:

(a) Physical risks (including any bodily contact or administration of any substance)? Yes  No

(b) Psychological/emotional risks (feeling uncomfortable, embarrassed, anxious or upset)? Yes  No

(c) Social risks (including possible loss of status, privacy and/or reputation)? Yes   No

(d) Is there any deception involved? (See Debriefing, #21) Yes   No

2. If you answered **Yes** to any of the above, please explain the risks, and describe how they will be managed and/or minimized.

Participation in the proposed research project will entail a substantial time commitment on the part of the college students. At times, they may feel a little overwhelmed or anxious. Prior to beginning the intervention, the principal investigator will be conducting an information session on campus whereby participants will be informed of what they can expect should they choose to participate. In addition Cogmed coaches, who are registered clinical psychologists, will be available to students throughout the duration of the study for extra support. These coaches will call the students weekly to check-in and see how they are managing. Calls will be made for all three groups who are involved in the training.

The neuroimaging tasks may take 60 minutes per session during which the participants are encouraged to restrict their movements. This extended time period of sitting still may be mildly uncomfortable. To minimize this discomfort, measures have been taken to allow for sufficient breaks during the testing procedure. It is also essential for the adequate recording of our measures that participants are in a comfortable and relaxed physical position as well as mental state. Therefore, at least once every 10 minutes there will be a scheduled break of at least 10 minutes long. Furthermore, the tasks will be programmed in a manner allowing for optional breaks at the participant’s convenience, or the researchers discretion, by simply pressing the spacebar.

The EGI sensor nets used for the brain measurements are amongst the most easy-to-apply, user-friendly and widely used methods of doing brain imaging. Due to its safe application procedures and noninvasive ways of measurement, the method is even used in infant research (Leppanen et al., 2007).

**18. POSSIBLE BENEFITS**

Discuss any potential direct benefits to the participants from their involvement in the project. Comment on the (potential) benefits to the scientific/scholarly community or society that would justify involvement of participants in this study.

Participants may gain direct benefit from participating in the project in terms of improvement in their working memory abilities and a possible decrease in their ADHD symptoms. Also, findings from this study will contribute to the growing data-base on the efficacy of working memory training in individuals with ADHD/LD. This information will in turn help other individuals with ADHD to understand the effectiveness and utility of working memory training. Upon request, an image of the participant’s brain activity will be mailed to the participant.

| **SECTION D – THE INFORMED CONSENT PROCESS** |
| --- |

**19. THE CONSENT PROCESS**

Describe the process that the investigator(s) will be using to obtain informed consent. Please include the experience of the team member with this participant population and/or training that this person will receive prior to recruitment. If there will be no written consent form, please explain (e.g. discipline, cultural appropriateness, etc.). Please note, it is the quality of the consent, not the format that is important. If the research involves extraction or collection of personal information from a data subject, please describe how consent from the individuals or authorization from the custodian will be obtained.

For information about the required elements in the information letter and consent form, please refer to [**http://www.research.utoronto.ca/ethics/eh_best.html**](http://www.research.utoronto.ca/ethics/eh_best.html)**.**

**Where applicable, please attach a copy of the Information Letter/Consent Form, the content of any telephone script, letters of administrative consent or authorization and/or any other material which will be used in the informed consent process.**

The primary and faculty investigators will be holding an information session on campus, where students will have the opportunity to ask questions. Those who are interested will be given the opportunity to sign up at that point and the consent procedures will be explained to them face-to-face. Should they choose to participate, written consent will be obtained (please see attached consent form for further information). A second session will also be held, for those individuals who may wish to sign up later.

**20. CONSENT BY AN AUTHORIZED PARTY**

If the participants are children, or are not competent to consent, describe the proposed alternate source of consent, including any permission/information letter to be provided to the person(s) providing the alternate consent as well as the assent process for participants.

N/A

**21. DEBRIEFING**

(a) If deception will be used in the research study, please explain what information/feedback will be provided to participants after participation in the project.

**Please provide a copy of the written debriefing form, if applicable.**

N/A

(b) How will participants be informed of study results?

Upon completing the study JVS psychologists will provide individual feedback on participants’ individual performance. Also, after the study has been completed, participants will be provided with a summary of the major findings from the study.

**22. PARTICIPANT WITHDRAWAL**

(a) Where applicable, please describe how the participants will be informed of their right to withdraw from the project. Outline the procedures which will be followed to allow them to exercise this right.

When written consent is obtained from the participants, they will be informed verbally about their right to withdraw from the project. Permission will also be requested to administer post-tests at the time of withdrawal. Should participants choose to exercise this right, they can contact Angela Zacharakis, Dr. Tannock’s administrative assistant (who is not involved in the project) or Karizma Mawjee (Project coordinator), either by phone or e-mail.

(b) Indicate what will be done with the participant’s data and any consequences which withdrawal may have on the participant.

If participants choose to withdraw at any point during the intervention, there will be no consequences. Their data will be kept in a filing cabinet under lock and key. Given that this is a randomized controlled trial, we aim to use an Intent-to-Treat with Last Observation Carried Forward approach (ITT-LOCF) in analyzing the data. Thus we will request permission to use the existing data from any participant who withdraws prematurely.

(c) If participants will not have the right to withdraw from the project at all, or beyond a certain point, please explain.

N/A

| **SECTION E –CONFIDENTIALITY AND PRIVACY** |
| --- |

**23. CONFIDENTIALITY**

(a) Will the data be treated as confidential? Yes  No

(b) Describe the procedures to be used to ensure anonymity of participants or informants, where applicable, or the confidentiality of data during the conduct of research and dissemination of results.

JVS is the licensed site that will be providing the Cogmed working memory training program. Participants’ daily progress is automatically logged to an electronic file, which is uploaded by a “training aide/coach” who is a licensed clinical psychologist or psychological associate, at the training site, JVS Toronto. Therefore, JVS will know the participants performance on Cogmed. Both the principal and co-investigators will know the participants’ summary scores on Cogmed as well as their scores on the various other measures. However, the college students themselves will not know their performance.

(c) Explain how written records, video/audio tapes and questionnaires will be secured, how long they will be retained, and provide details of their final disposal or storage.

All written data collected will be safely stored in a locked cabinet in a locked research office in HDAP at OISE. All participants’ names will be stripped from all documents and replaced with ID codes. The primary investigator will hold the file linking ID’s to names in a separate locked cabinet. All data will be archived for the required time period after completion of the study.

(d) If participant anonymity or confidentiality is not appropriate to this research project, please explain.

N/A

**24. PRIVACY REGULATIONS**

For research involving extraction or collection of personal information, provincial, national and/or international laws may apply. **My signature as Principal Investigator, in Section G of this protocol form, confirms that I understand and will comply with all relevant laws governing the collection and use of personal information in research.**

| **SECTION F – CONTINUING REVIEW OF ONGOING RESEARCH** |
| --- |

**RISK MATRIX: REVIEW TYPE BY GROUP VULNERABILITY AND RESEARCH RISK – check one:**

**Research Risk**

**Group Vulnerability Low Medium High**

**Low** **1**  **1**  **2**

**Medium** **1**  **2**  **3**

**High** **2**  **3**  **3**

See the *Instructions for Ethics Review Protocol Submission Form* for detailed information about the Risk Matrix.

Briefly explain/justify the level of risk and group vulnerability reported above (max 100 words):

College students with ADHD/LD are young adults who face various struggles (academic, social etc.) and many of whom are living away from home. Given the intensity of the training program, many of these students may feel overwhelmed and anxious at times and some of these students may feel they are lacking necessary supports to cope. The registered psychologists and psychological associates at JVS (‘training coaches’) will be able to help these students manage their stress and balance the demands of the program with the demands of their daily life.

**Review Type**

Based on the level of risk, please submit the appropriate number of copies of the Protocol Submission Form for Review Type:

**Risk level = 1: Expedited Review Risk level = 2 or 3: Full Review**

Information about individual REBs, including the number of copies required for each review type, can be found here: [**www.research.utoronto.ca/ethics/eh_rebs.html**](file:///C:\Documents%20and%20Settings\Steven%20Woltering\My%20Documents\_Projects\COGMED%20Rosemary\Ethics\Amendment%20CIHR%202012\www.research.utoronto.ca\ethics\eh_rebs.html)

**Please note that the final determination of Review Type and program of Continuing Review will be made by the University of Toronto REB and the Ethics Review Office.**

| **SECTION G – SIGNATURES** |
| --- |

**All researchers and their respective Departmental Chair/Dean or designate must sign below:**

As the **Investigator** on this project, my signature confirms that I will ensure that all procedures performed under the project will be conducted in accordance with all relevant University, provincial, national and international policies and regulations that govern research involving human participants. Any deviation from the project as originally approved will be submitted to the Research Ethics Board for approval prior to its implementation.

For **student researchers**, my signature confirms that I am a registered student in good standing with the University of Toronto. My project has been reviewed and approved by my advisory committee (where applicable). If my status as a student changes, I will inform the Ethics Review Office.

| Signature of Investigator: 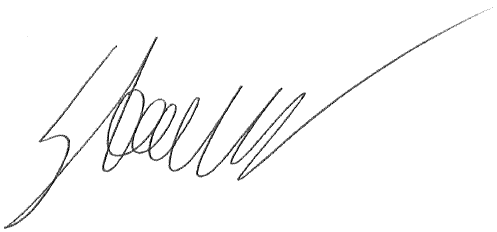 Date: Jan 4, 2012 |
| --- |

**For Graduate Students the signature of the Faculty Supervisor is required. For Post-Doctoral Fellows and Visiting Professors or Researchers, the signature of the Faculty Sponsor is required.**

As the **Faculty Supervisor** of this project, my signature confirms that I have reviewed and approve the scientific merit of the research project and this ethics protocol submission. I will provide the necessary supervision to the student researcher throughout the project, to ensure that all procedures performed under the research project will be conducted in accordance with relevant University, provincial, national or international policies and regulations that govern research involving human subjects. This includes ensuring that the level of risk inherent to the project is managed by the level of research experience that the student has, combined with the extent of oversight that will be provided by the Faculty Supervisor and/or On-site Supervisor.

As the **Faculty Sponsor** for this project, my signature confirms that I have reviewed and approve of the research project and will assume responsibility, as the University representative, for this research project. I will ensure that all procedures performed under the project will be conducted in accordance with all relevant University, provincial, national or international policies and regulations that govern research involving human participants.

| Signature of Faculty Supervisor/Sponsor: 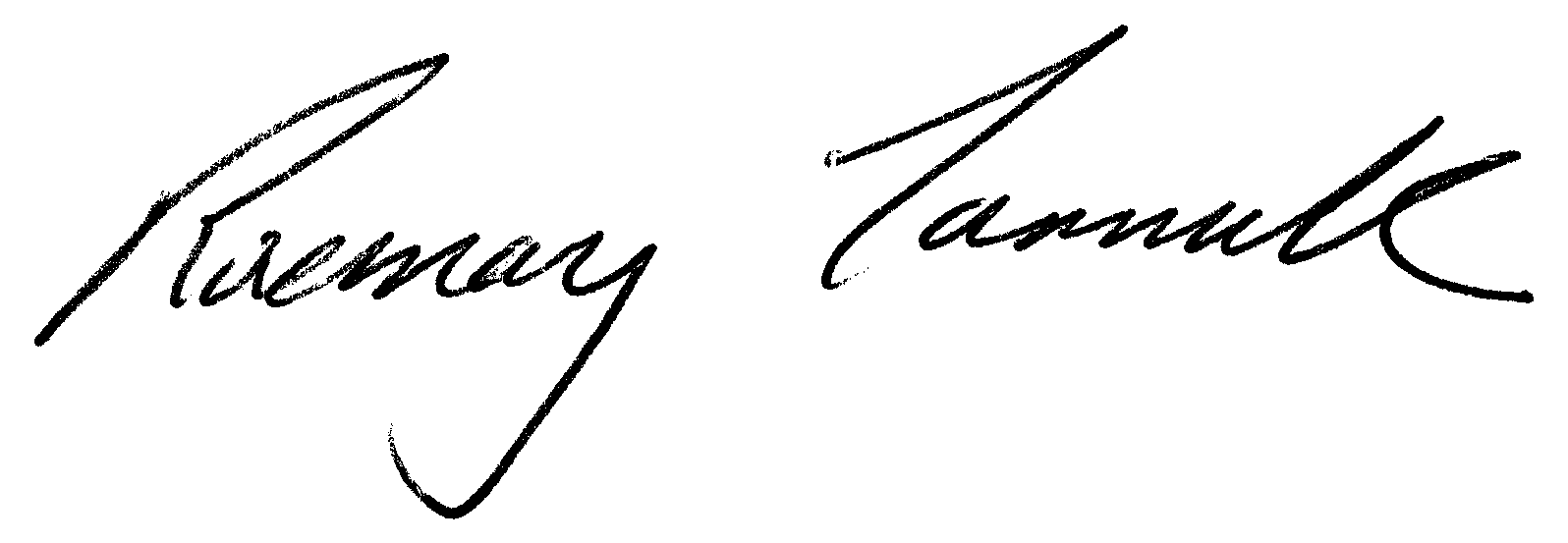 Date: Jan 4, 2012 |
| --- |

As the **Departmental Chair/Dean**, my signature confirms that I am aware of the proposed activity. My administrative unit will follow guidelines and procedures which ensure compliance with all relevant University, provincial, national or international policies and regulations that govern research involving human subjects. My signature also reflects the willingness of the department, faculty or division to administer the research funds, if there are any, in accordance with University, regulatory agency and sponsor agency policies.

| Name of Departmental Chair/Dean (or designate): Esther Geva  Signature of Departmental Chair/Dean: 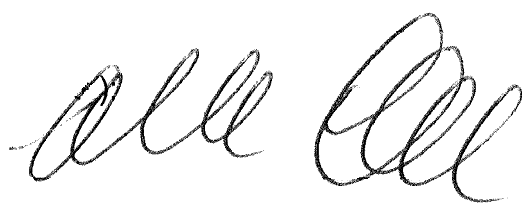 Date: Jan 6, 2012  (or designate) |
| --- |

1. Polanczyk G, Jensen P. Epidemiologic considerations in attention deficit hyperactivity disorder: a review and update. Child Adolesc Psychiatr Clin N Am. [Research Support, Non-U.S. Gov't

Review]. 2008 Apr;17(2):245-60, vii.

2. Shaywitz SE, Shaywitz BA, Fletcher JM, Escobar MD. Prevalence of reading disability in boys and girls. Results of the Connecticut Longitudinal Study. Jama. [Research Support, Non-U.S. Gov't

Research Support, U.S. Gov't, Non-P.H.S.

Research Support, U.S. Gov't, P.H.S.]. 1990 Aug 22-29;264(8):998-1002.

3. Sexton CC, Gelhorn H, Bell J, Classi P. The Co-occurrence of Reading Disorder and ADHD: Epidemiology, Treatment, Psychosocial Impact, and Economic Burden. J Learn Disabil. 2011 Jul 14.

4. McGrath LM, Pennington BF, Shanahan MA, Santerre-Lemmon LE, Barnard HD, Willcutt EG, et al. A multiple deficit model of reading disability and attention-deficit/hyperactivity disorder: searching for shared cognitive deficits. J Child Psychol Psychiatry. [Research Support, N.I.H., Extramural]. 2011 May;52(5):547-57.

5. Willcutt EG, Betjemann RS, McGrath LM, Chhabildas NA, Olson RK, DeFries JC, et al. Etiology and neuropsychology of comorbidity between RD and ADHD: the case for multiple-deficit models. Cortex. [Twin Study]. 2010 Nov-Dec;46(10):1345-61.

6. Baddeley A. Working memory: theories, models, and controversies. Annu Rev Psychol. 2012 Jan 10;63:1-29.

7. Alloway TP, Gathercole SE, Elliott J. Examining the link between working memory behaviour and academic attainment in children with ADHD. Dev Med Child Neurol. [Research Support, Non-U.S. Gov't]. 2010 Jul;52(7):632-6.

8. Alloway TP, Gathercole SE, Kirkwood H, Elliott J. The cognitive and behavioral characteristics of children with low working memory. Child Dev. [Research Support, Non-U.S. Gov't]. 2009 Mar-Apr;80(2):606-21.

9. Gathercole SE, Alloway TP, Willis C, Adams AM. Working memory in children with reading disabilities. J Exp Child Psychol. [Research Support, Non-U.S. Gov't]. 2006 Mar;93(3):265-81.

10. Klingberg T. Training and plasticity of working memory. Trends Cogn Sci. [Review]. 2010 Jul;14(7):317-24.

11. Westerberg H, Jacobaeus H, Hirvikoski T, Clevberger P, Ostensson ML, Bartfai A, et al. Computerized working memory training after stroke--a pilot study. Brain Inj. [Randomized Controlled Trial

Research Support, Non-U.S. Gov't]. 2007 Jan;21(1):21-9.

12. Westerberg H, Hirvikoski T, Forssberg H, Klingberg T. Visuo-spatial working memory span: a sensitive measure of cognitive deficits in children with ADHD. Child Neuropsychol. [Comparative Study

Research Support, Non-U.S. Gov't]. 2004 Sep;10(3):155-61.

13. Beck SJ, Hanson CA, Puffenberger SS, Benninger KL, Benninger WB. A controlled trial of working memory training for children and adolescents with ADHD. J Clin Child Adolesc Psychol. [Multicenter Study

Randomized Controlled Trial]. 2010;39(6):825-36.

14. Brehmer Y, Rieckmann A, Bellander M, Westerberg H, Fischer H, Backman L. Neural correlates of training-related working-memory gains in old age. Neuroimage. [Research Support, Non-U.S. Gov't]. 2011 Oct 15;58(4):1110-20.

15. Holmes J, Gathercole SE, Dunning DL. Adaptive training leads to sustained enhancement of poor working memory in children. Dev Sci. [Research Support, Non-U.S. Gov't]. 2009 Jul;12(4):F9-15.

16. Westerberg H, Klingberg T. Changes in cortical activity after training of working memory--a single-subject analysis. Physiol Behav. 2007 Sep 10;92(1-2):186-92.

17. Olesen PJ, Westerberg H, Klingberg T. Increased prefrontal and parietal activity after training of working memory. Nat Neurosci. [Comparative Study

Research Support, Non-U.S. Gov't]. 2004 Jan;7(1):75-9.

18. Woodcock R, McGrew K., Mather, N. Woodcock Johnson III Tests of Cognitive Abilities (WJ III COG): Riverside; 2001.

19. Wechsler D. Wechsler Abbreviated Scales of Intelligence. . San Antonio, TX: Psychological Corp; 1999.

20. Adler LA, Spencer T, Faraone SV, Kessler RC, Howes MJ, Biederman J, et al. Validity of pilot Adult ADHD Self- Report Scale (ASRS) to Rate Adult ADHD symptoms. Ann Clin Psychiatry. 2006 Jul-Sep;18(3):145-8.

21. Murphy KR, Adler LA. Assessing attention-deficit/hyperactivity disorder in adults: focus on rating scales. J Clin Psychiatry. 2004;65 Suppl 3:12-7.

22. Kinnear PR, Sahraie A. New Farnsworth-Munsell 100 hue test norms of normal observers for each year of age 5-22 and for age decades 30-70. Br J Ophthalmol. 2002 Dec;86(12):1408-11.

23. Mollon JD RJ. Manual for the Mollon-Reffin Minimalist Test. Version 0.7. 1994.

24. Klingberg T, Fernell E, Olesen PJ, Johnson M, Gustafsson P, Dahlstrom K, et al. Computerized training of working memory in children with ADHD--a randomized, controlled trial. J Am Acad Child Adolesc Psychiatry. 2005 Feb;44(2):177-86.

25. Westerberg H, Jacobaeus H, Hirvikoski T, Clevberger P, Ostensson ML, Bartfai A, et al. Computerized working memory training after stroke--a pilot study. Brain Inj. 2007 Jan;21(1):21-9.

26. Wechsler D, Kaplan, E., Fein, D., Kramer, J., Morris, R., Delis, D., Maerlander, A. Wchsler Intelligence Scale for Children - Fourth Ecition, Integrated (WISC-IV Integrated): The Psychological Corporation; 2004.

27. Tursky B SD, Crider A, & Kahneman D

Pupillary, heart rate, and skin resistance changes during a mental task. . J Exp Psychol Hum Percept Perform. 1969;79(1):164-7.

28. Fray PJ RT, Sahakian BJ. Neuropsychiatric applications of CANTAB. International Journal of Geriatric

Psychology. 1996;11:329-36.

29. Adams W SD. Wide Range Assessment of Memory and Learning. . Wilmington (Delaware): Wide Range Inc.; 1990.

30. Petrides M, Milner B. Deficits on subject-ordered tasks after frontal- and temporal-lobe lesions in man. Neuropsychologia. 1982;20(3):249-62.

31. Diamond A, Briand L, Fossella J, Gehlbach L. Genetic and neurochemical modulation of prefrontal cognitive functions in children. Am J Psychiatry. 2004 Jan;161(1):125-32.

32. Espinosa A, Alegret M, Boada M, Vinyes G, Valero S, Martinez-Lage P, et al. Ecological assessment of executive functions in mild cognitive impairment and mild Alzheimer's disease. J Int Neuropsychol Soc. 2009 Sep;15(5):751-7.

33. Wilson BA AN, Burgess PW, Emslie H, Evans JJ. . The behavioural assessment of the dysexecutive syndrome. . Bury St Edmunds: Thames Valley Company; 1996.

34. Broadbent DE, Cooper PF, FitzGerald P, Parkes KR. The Cognitive Failures Questionnaire (CFQ) and its correlates. Br J Clin Psychol. 1982 Feb;21 (Pt 1):1-16.

35. Barkley RA, Murphy KR. Impairment in occupational functioning and adult ADHD: the predictive utility of executive function (EF) ratings versus EF tests. Arch Clin Neuropsychol. [Comparative Study

Research Support, N.I.H., Extramural]. 2010 May;25(3):157-73.

36. Barkley RA. Barkley Deficits in Executive Functioning Scale (BDEFS). New York: Guilford; 2011.
